# Deciphering causal and statistical relations of molecular aberrations and gene expressions in NCI-60 cell lines

**DOI:** 10.1186/1752-0509-5-186

**Published:** 2011-11-04

**Authors:** Shyh-Dar Li, Tatsuaki Tagami, Ying-Fu Ho, Chen-Hsiang Yeang

**Affiliations:** 1 Institute of Statistical Science, Academia Sinica, Academia Road, Sec 2, Taipei, Taiwan; 2 Ontario Institute for Cancer Research, 101 College Street, Toronto, Canada; 3 Nagoya City University, Nagoya, Japan

## Abstract

**Background:**

Cancer cells harbor a large number of molecular alterations such as mutations, amplifications and deletions on DNA sequences and epigenetic changes on DNA methylations. These aberrations may dysregulate gene expressions, which in turn drive the malignancy of tumors. Deciphering the causal and statistical relations of molecular aberrations and gene expressions is critical for understanding the molecular mechanisms of clinical phenotypes.

**Results:**

In this work, we proposed a computational method to reconstruct *association modules *containing driver aberrations, passenger mRNA or microRNA expressions, and putative regulators that mediate the effects from drivers to passengers. By applying the module-finding algorithm to the integrated datasets of NCI-60 cancer cell lines, we found that gene expressions were driven by diverse molecular aberrations including chromosomal segments' copy number variations, gene mutations and DNA methylations, microRNA expressions, and the expressions of transcription factors. In-silico validation indicated that passenger genes were enriched with the regulator binding motifs, functional categories or pathways where the drivers were involved, and co-citations with the driver/regulator genes. Moreover, 6 of 11 predicted MYB targets were down-regulated in an MYB-siRNA treated leukemia cell line. In addition, microRNA expressions were driven by distinct mechanisms from mRNA expressions.

**Conclusions:**

The results provide rich mechanistic information regarding molecular aberrations and gene expressions in cancer genomes. This kind of integrative analysis will become an important tool for the diagnosis and treatment of cancer in the era of personalized medicine.

## Background

Cancer cells harbor a large number of alterations at genetic, epigenetic and phenotypic levels. High-throughput screenings identified hundreds of somatic mutations (e.g., [1,2]), thousands of gene and microRNA expression changes (e.g., [3,4]) and copy number variations (e.g., [5]), large-scale epigenetic variations (e.g., [6]) in a typical cancer cell. Only a small fraction of these alterations may drive the malignancy of cancers, whereas the majority of them are likely the by-products of chromatin instability and dysregulation of transcriptional/translational apparatus. Separation of driver from passenger aberrations is a key question of cancer genomics for its strong implications in prognosis and treatments.

Finding the causal and mechanistic links connecting driver aberrations and clinical phenotypes is challenging due to the complexity of the underlying processes. Alternatively, molecular phenotypes such as gene expressions are considered. Genetic and epigenetic alterations on DNAs modulate the expressions and activities of key regulators such as transcription factors and microRNAs. Dysregulation of these molecules in turn affects a large number of downstream genes. The global gene expression and activity changes then influence clinical phenotypes such as proliferation rates, drug resistance and capability of metastasis.

Within the context of mechanisms we modify and extend the definitions of drivers and passengers. A driver is a molecular aberration (mutation, copy number variation, DNA methylation, etc.) that causes variations of gene expressions. A passenger is a protein-coding gene or microRNA whose expression is modulated by the driver molecular aberration. Notice our definitions of drivers and passengers specify mechanistic/causal relations of genes but do not require information about clinical phenotypes. We term the conventional definitions of drivers and passengers as phenotypic and the extended definitions as mechanistic. A phenotypic driver is usually also a mechanistic driver, since it may affect clinical phenotypes by altering the regulatory programs in cancer cells. Conversely, a mechanistic driver may not be a phenotypic driver if the altered passenger genes do not pertain to the clinical phenotypes. Similarly, a mechanistic passenger can be a phenotypic driver if the expression change of the gene affects the cancer phenotype.

Comprehensive characterization of diverse molecular aberrations in tumors is a major trend of cancer research in the post-genomic era. Several international consortia and research institutions have launched large-scale projects to catalog the genomic, transcriptomic and epigenetic changes across multiple tumor types. Two noted endeavors are The Cancer Genome Atlas (TCGA) [7] and the International Cancer Genome Consortium (ICGC) [8]. Beyond these coordinated efforts, comprehensive assays on the NCI-60 cell lines have been performed by distinct research groups over the last two decades (e.g., [9-19]).

As large-scale, comprehensive cancer genomic data become more abundant, it is essential to develop computational tools to integrate heterogeneous data in order to acquire a systematic understanding of cancer cells. A rich collection of previous studies attempted to identify the driver mutations responsible for tumorigenesis from cancer genomic data (e.g., [2,20]). These studies hypothesized that the driver mutations conferred selective advantages for tumor growth thus occurred in multiple independent instances. Accordingly they aimed for finding recurrent aberrations with high frequencies. Beyond single genes a variety of computational tools have been proposed to examine the abnormal pathway activities by combining the molecular information of their constituent genes, such as the gene set enrichment analysis [21], principal component analysis [22], factor graph models [23], and others (e.g., [24]). Causal and mechanistic relations of molecular aberrations and gene expressions were not addressed in these studies. Some studies tracked the causes of abnormal gene expressions by correlating them with DNA copy numbers, gene mutations, DNA methylations or microRNA expressions (e.g., [7,25-27,4]). However, these studies were often restricted to pairwise comparisons between two types of data and lacked a unifying framework to integrate multiple types of data in the same model.

Beyond cancer data analysis many generic computational models of data integration have been proposed. Causal relations were constrained by a variety of properties such as conditional independence of observed variables (e.g., [28,29]), molecular interactions and pathways (e.g., [30]), and data generated from intervention experiments (e.g., [31]). While many of these tools have been applied to cancer data, most of the questions of interest are phenotype-driven (e.g., finding genes responsible for a clinical trait) rather than mechanism-driven (e.g., finding the causal relations connecting driver aberrations and passenger gene expressions).

Akavia et al. inferred mechanistic relations of gene expressions and phenotypes in melanoma [32]. They identified the gene copy number changes that modulated their own expressions and indirectly affected the expressions of their target genes. Curiously, these mechanistic drivers were also phenotypic drivers, as their knock-downs abrogated tumor cell proliferation. Despite the value of connecting mechanistic and phenotypic characterization, this study was restricted to copy number variations and discarded other types of molecular aberrations.

Recently, we proposed a layered modeling framework for integrative analysis of cancer genomic data [33]. The goal was to explain gene expressions with observed molecular aberrations. Associations with molecular aberrations were incrementally included according to levels of uncertainty and mechanistic information. In this work, we extended and modified the layered modeling framework to identify association modules of driver molecular aberrations and passenger gene/mircroRNA expressions from the integrated datasets of NCI-60 cell lines. For each type of molecular aberrations, we found the downstream passengers putatively affected by the drivers and regulators mediating the effects from drivers to passengers. The causal relations between drivers/regulators and passengers were supported by both experimental and in-silico validations. The analysis results justify the utility of association modules for other cancer genomic data.

## Results

### Construction of association modules from integrated datasets

Diverse molecular aberrations can modulate variations of gene expressions in cancer cells. The dependencies between molecular aberrations and gene expressions may be modular, as multiple genes with coherent expression profiles are likely to be affected by common molecular aberrations. The goal of this study is to find the modules of genes or microRNAs whose coherent expression profiles are possibly driven by common molecular aberrations. We define an association module as a tuple consisting of three components: (1)observed driver molecular aberrations, (2)passen-ger genes or microRNAs whose expression profiles are associated with driver molecular aberrations, (3)regulators (transcription factors) that mediate the effects between drivers and passengers. We consider the following types of association modules:

1. *Cis-acting effects with copy number variations of chromosomal segments*. The copy number variation (CNV) of a chromosomal segment is associated with the expressions of its constituent genes or microRNAs.

2. *Trans-acting effects with copy number variations of chromosomal segments*. A chromosomal segment CNV possesses cis-acting effects with intermediate regulators on the segment, and both segment CNV and regulator expressions are associated with the expressions of genes or microRNAs on other segments.

3. *Effects with gene mutations*. The mutational state of a gene is associated with the expressions of itself and other genes or microRNAs.

4. *Effects with DNA methylations*. The coherent DNA methylation state of a collection of genes is negatively associated with the expressions of genes or microRNAs.

5. *Regulatory effects with microRNAs*. The coherent expressions of a collection of microRNAs are negatively associated with the expressions of a collection of genes.

6. *Regulatory effects with transcription factors*. The coherent expressions of a collection of transcription factors are associated with the expressions of a collection of other genes.

**Figure 1:**
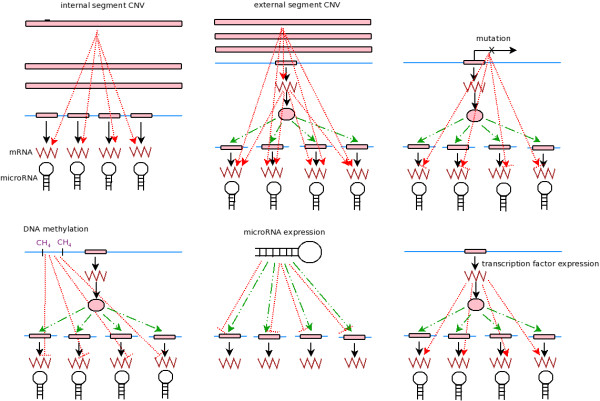
**Types of association modules**. From top-left to bottom-right: cis-acting effects with copy number variations of chromosomal segments, trans-acting effects with copy number variations of chromosomal segments, associations with gene mutations, associations with DNA methylations, regulatory effects with microRNAs, regulatory effects with transcription factors. Black solid lines: information flows from central dogma. Green dashed lines: regulatory links from transcription factors or microRNAs to their targets on other chromosomal locations. Red dotted lines: associations between observed aberrations and mRNA/microRNA expressions. Arrows indicate positive associations and bar-ends indicate negative associations.

The association modules are visually summarized in Figure 1 and elaborated in Materials and Methods and Additional file 1, Text S1.

Associations between driver aberrations and passenger expressions are established by a series of hypothesis testing procedures using logistic regression models. In brief, an association between a driver and a passenger is selected only if it cannot be replaced by any other association (with the same passenger) without a significant loss of the explanatory power. Procedures of constructing association modules are described in Materials and Methods and Additional file 1, Text S1.

### Diverse molecular aberrations drive mRNA responses in NCI-60 cell lines

We identified association modules from integrated datasets of NCI-60 cell lines, including mRNA and microRNA expressions, copy number variations, mutations, and DNA methylations (see Materials and Methods for the data sources and processing). 6888 genes possessed coherent expression profiles among three mRNA datasets. 48.16% (3317 of 6888) of the valid mRNA expression profiles were explained by observed molecular aberrations. Intriguingly, each type of association modules in Figure 1 were detected, suggesting diverse molecular mechanisms drive mRNA expressions.

#### Summary of mRNA association modules

Table 1 shows the summary information of association modules for mRNA expressions. Associations with segment CNVs dominate the inferred modules. 1699 of 3317 passenger expressions explained by observed aberrations are associated with CNVs on their own segments, and 43 of 84 association modules are cis-acting effects with segment CNVs. Trans-acting effects with segment CNVs comprise 18 association modules and 506 passenger genes. 522 passenger expressions and 14 association modules are positively or negatively associated with mutations of 8 oncogenes and tumor suppressors. 161 passenger expressions and 5 association modules are negatively associated with DNA methylations. Two association modules with microRNA expressions comprise 121 and 120 passenger genes respectively. Moreover, two association modules with transcription factor expressions comprise 338 and 338 passenger genes respectively. The complete list of association modules are reported in Additional file 2, Table S1, and driver aberrations and passenger mRNA expressions are visualized in Additional file 1, Figures S1, S2, S3, S4, S5, S6, S7, S8, S9, S10, S11, S12 and S13. The complete list of microRNA association modules are reported in Additional file 3, Table S2, and driver aberrations and passenger microRNA expressions are visualized in Additional file 1, Figures S14, S15, S16.

**Table 1:** Summary information of association modules for mRNA expressions

index	driver type	drivers	reg	sign	*N*	index	driver type	drivers	reg	sign	*N*
1	intra CNV	seg 30 (chr 6p)	NA	+	84	44	inter CNV	seg 18 (chr 3p)	ZIC1,MITF	+	118
2	intra CNV	seg 54 (chr 12q)	NA	+	77	45	inter CNV	seg 31 (chr 6q)	TBP,MYB,HDAC2	+	77
3	intra CNV	seg 1 (chr 1p)	NA	+	74	46	inter CNV	seg 84 (chr 22q)	SREBF2,EP300	+	49
4	intra CNV	seg 57 (chr 14q)	NA	+	62	47	inter CNV	seg 30 (chr 6p)	E2F3,NFYA,SRF	+	31
5	intra CNV	seg 69 (chr 17q)	NA	+	62	48	inter CNV	seg 42 (chr 10q)	TCF7L2	+	30
6	intra CNV	seg 11 (chr 2p)	NA	+	58	49	inter CNV	seg 55 (chr 12q)	NR2C1,NFYB	+	22
7	intra CNV	seg 42 (chr 10q)	NA	+	55	50	inter CNV	seg 19 (chr 3q)	ZNF148	+	22
8	intra CNV	seg 3 (chr 1p)	NA	+	53	51	inter CNV	seg 63 (chr 16pq)	NFATC3,RBL2	+	21
9	intra CNV	seg 37 (chr 8q)	NA	+	53	52	inter CNV	seg 69 (chr 17q)	SPOP,NME2,POLG2	+	20
10	intra CNV	seg 59 (chr 14q)	NA	+	53	53	inter CNV	seg 42 (chr 10q)	NFKB2	+	19
11	intra CNV	seg 16 (chr 3p)	NA	+	49	54	inter CNV	seg 16 (chr 3p)	UBP1	+	18
12	intra CNV	seg 63 (chr 16pq)	NA	+	49	55	inter CNV	seg 83 (chr 22q)	EWSR1	+	17
13	intra CNV	seg 33 (chr 7q)	NA	+	47	56	inter CNV	seg 57 (chr 14q)	APEX1	+	16
14	intra CNV	seg 84 (chr 22q)	NA	+	47	57	inter CNV	seg 16 (chr 3p)	RPA2,EIF2C1	+	15
15	intra CNV	seg 55 (chr 12q)	NA	+	44	58	inter CNV	seg 19 (chr 3q)	CNBP	+	15
16	intra CNV	seg 51 (chr 11q)	NA	+	43	59	inter CNV	seg 51 (chr 11q)	RICS	+	14
17	intra CNV	seg 2 (chr 1p)	NA	+	42	60	inter CNV	seg 56 (chr 13q)	GTF3A	+	13
18	intra CNV	seg 73 (chr 19p)	NA	+	42	61	inter CNV	seg 4 (chr 1q)	RUNX3	+	10
19	intra CNV	seg 50 (chr 11q)	NA	+	40	62	mutation	APC	NA	+	136
20	intra CNV	seg 68 (chr 17q)	NA	+	39	63	mutation	APC	NA	-	56
21	intra CNV	seg 65 (chr 17p)	NA	+	37	64	mutation	PIK3CA	NA	+	50
22	intra CNV	seg 32 (chr 7p)	NA	+	36	65	mutation	CDKN2A	NA	-	43
23	intra CNV	seg 35 (chr 7q)	NA	+	35	66	mutation	TP53	NA	-	35
24	intra CNV	seg 31 (chr 6q)	NA	+	33	67	mutation	BRAF	NA	-	34
25	intra CNV	seg 19 (chr 3q)	NA	+	32	68	mutation	TP53	NA	+	33
26	intra CNV	seg 18 (chr 3p)	NA	+	30	69	mutation	CDKN2A	NA	+	32
27	intra CNV	seg 9 (chr 1q)	NA	+	29	70	mutation	KRAS	NA	+	25
28	intra CNV	seg 22 (chr 4p)	NA	+	29	71	mutation	PTEN	NA	+	20
29	intra CNV	seg 62 (chr 15q)	NA	+	29	72	mutation	KRAS	NA	-	16
30	intra CNV	seg 41 (chr 9q)	NA	+	28	73	mutation	BRAF	NA	+	16
31	intra CNV	seg 49 (chr 11q)	NA	+	28	74	mutation	PIK3CA	NA	-	13
32	intra CNV	seg 56 (chr 13q)	NA	+	28	75	mutation	PTEN	NA	-	13
33	intra CNV	seg 60 (chr 15q)	NA	+	28	76	methylation	PAX8	NA	-	61
34	intra CNV	seg 44 (chr 11p)	NA	+	27	77	methylation	BCR	NA	-	58
35	intra CNV	seg 61 (chr 15q)	NA	+	26	78	methylation	HOXC13	NA	-	19
36	intra CNV	seg 80 (chr 21q)	NA	+	24	79	methylation	CCND1,PPARG	NA	-	13
37	intra CNV	seg 36 (chr 8p)	NA	+	23	80	methylation	COL5A1	NA	-	10
38	intra CNV	seg 27 (chr 5q)	NA	+	22	81	microRNA	mir group 1	NA	-	121
39	intra CNV	seg 39 (chr 9p)	NA	+	22	82	microRNA	mir group 2	NA	-	120
40	intra CNV	seg 12 (chr 2q)	NA	+	20	83	TF	TF group 1	NA	+	338
41	intra CNV	seg 74 (chr 19q)	NA	+	20	84	TF	TF group 2	NA	+	338
42	intra CNV	seg 83 (chr 22q)	NA	+	20						
43	intra CNV	seg 85 (chr 23pq)	NA	+	20						

Index: module index. Reg: intermediate regulators. Sign: the functional direction (activation or repression) from drivers to passengers. *N*: number of passenger genes. Intra CNV: intra-segment CNV. Inter CNV: inter-segment CNV. TF: transcription factor expressions. The locations of segments are reported in Supplementary Table S5. Mir group 1 consists of mir-92,mir-96,mir-106a,mir 20b,mir-17,mir-19b,mir-32,mir-135,mir-25,mir-106b,mir-93,mir-106,mir-18,mir-20. Mir group 2 consists of mir-24,mir-99b,mir-27b,mir-21,mir-125a,mir-23b,mir-27a,mir-23a. TF group 1 consists of SMAD3,FOXD1,PLAU,BDNF,FOSL2,FOSL1,RBMS1. TF group 2 consists of ERG,ELK4,NFATC1,RFX3,POU5F1,ZNF350.

The credibility of associations between multiple variables was assessed by false discovery rates (FDR, [34,35]). We adopted the permutation tests described in [36] as the null model and evaluated two types of FDRs:

$\left(1\right)\;\frac{\text{expected \# false positives according to the null model}}{\#\text{positive calls from the date}},\;\left(2\right)\;\frac{\#\text{ false positives in the 99 percentile of the null model}}{\#\text{ positive calls from the date}}$ Table 2 shows the FDRs for each type of associations and for all associations together. The FDRs for cis and trans acting effects with segment CNVs are substantially smaller than those for other types of associations. This is sensible as the cis-acting effects with segment CNVs are constrained by chromosomal locations of passenger genes, and the trans-acting effects with segment CNVs are constrained by coherent segment CNVs and expressions of their regulators. In contrast, other types of associations possess no additional constraints beyond driver aberrations and passenger expressions, thus are more likely to be spurious. Furthermore, the FDRs calculated from the expected number of false positives are lower than those calculated from the 99 percentile of the null distribution. This is also sensible since the latter gives a much more conservative estimate of the false positive numbers. The overall FDRs calculated by these two methods are 0.235 and 0.326 respectively.

To justify the biological meanings of association modules, we conducted several in-silico validations based on prior literature. First, we extracted putative targets of transcription factors from the TRANSFAC database [37] and those of microRNAs from three databases ([38-40]). For each association module, we then evaluated the enrichment p-values for the putative targets of their regulators (transcription factors or microRNAs) in the passenger genes. Table 3 reports the enrichment p-values for the putative targets of regulators in the association modules. Among the 14 modules whose regulators possess putative target information, 9 of them are significantly enriched (p-value < 0.05) with putative targets in their passenger genes. All but one modules containing more than 60 passenger genes are enriched with putative targets of their regulators. Only one large module - positive associations with APC mutations - is not enriched with the putative targets of the regulator since APC is not a transcription factor and has no binding motifs.

**Table 2:** False discovery rates of mRNA and microRNA association modules

**mRNA modules**:		
FDR evaluation	driver type	FDR
mean	intra CNV	0.0259
mean	inter CNV	0.0035
mean	mutation	0.3467
mean	methylation	0.4334
mean	microRNA	0.2248
mean	TF	0.2197
mean	all	0.2353
99%	intra CNV	0.0855
99%	inter CNV	0.0899
99%	mutation	0.4949
99%	methylation	0.6322
99%	microRNA	0.3109
99%	TF	0.2815
99%	all	0.3262
		
microRNA modules:		
FDR evaluation	driver type	FDR

mean	intra CNV	NA
mean	inter CNV	0.0021
mean	mutation	0.2535
mean	methylation	0.4403
mean	microRNA	NA
mean	TF	0.2857
mean	all	0.2924
99%	intra CNV	NA
99%	inter CNV	0.0455
99%	mutation	0.3645
99%	methylation	0.6379
99%	microRNA	NA
99%	TF	0.3600
99%	all	0.3943

Mean: using the expected number of false positives to evaluate FDR. 99%: using the 99 percentile of the number of false positives to evaluate FDR. Intra CNV: intra-segment CNV. Inter CNV: inter-segment CNV. TF: transcription factor expressions. All: the FDR over all associations.

**Table 3:** Enrichment of driver/regulator binding motifs on passenger promoters of mRNA association modules

index	reg	***N***_**1**_	***N***_**2**_	***N***_**3**_	*p-value*
44	MITF	118	2023	23	0.0464
45	TBP	77	8009	56	5.3704 × 10^-4^
45	MYB	77	10684	65	0.0076
46	SREBF2	49	9755	36	0.1581
47	E2F3	31	8123	21	0.0989
47	NFYA	31	2170	3	0.8513
47	SRF	31	75	0	1.0
51	NFATC3	22	2170	5	0.2097
53	NFKB2	19	531	0	1.0
66	TP53	35	3576	16	0.0041
76	PAX8	61	5376	34	0.0014
78	HOXC13	19	5421	12	0.0164
79	PPARG	13	20	0	1.0
81	mir-96	121	636	11	0.0147
81	mir-106a	121	319	6	0.0463
81	mir-17	121	830	12	0.0379
81	mir-93	121	832	12	0.0385
81	mir-106	121	718	11	0.0323
82	mir-21	120	291	7	0.0093
83	SMAD3	338	10025	271	1.058 × 10^-7^
84	ERG	338	2231	67	0.0093
84	ELK4	338	2803	76	0.0518
84	NFATC1	338	10187	265	3.1517 × 10^-5^
84	RFX3	338	2751	75	0.0481
84	POU5F	338	379	18	0.0027

Index: module index. Reg: the transcription factor or microRNA with putative targets. *N*_1_: number of passenger genes in a module. *N*_2_: total number of putative targets in human genes. *N*_3_: number of passenger genes containing the transcription factor binding motif. p-value: hyper-geometric p-value of enrichment. For modules with microRNAs as drivers, *N*_2_: number of putative targets of a microRNA. *N*_3_: number of passenger genes belonging to the putative targets of the microRNA.

Second, we incurred a batch search for all pairs of drivers/regulators and passengers of association modules on NCBI PubMed database and checked whether they were co-cited in previous publications. Table 4 summarizes the information of co-cited (driver/regulator, passenger) pairs for each association module. In total, 449 (driver/regulator, passenger) pairs co-occur in the same publications, and 22 of 46 association modules with non-local aberrations have co-cited (driver/regulator, passenger) pairs. To assess the significance of co-citations we randomly sampled passenger genes of each module and counted the maximum number of co-cited pairs over 10 random trials. Among the 23 association modules possessing co-cited pairs, 7 of them have more than 1.4 fold of the number of co-cited pairs compared to random samples (Table 4). The complete co-cited (driver/regulator, passenger) pairs among all association modules are reported in Additional file 4, Table S3.

**Table 4:** Summary of co-citations in mRNA and microRNA association modules

mRNA modules				
index	drivers/regulators	***N***_**1**_	***N***_**2**_	***N***_**3**_
44	ZIC1,MITF seg 18 CNV (+)	118	14	6
45	TBP,MYB,HDAC2 seg 31 CNV (+)	77	28	20
46	SREBF2,EP300 seg 84 CNV (+)	49	3	2
47	E2F3,NFYA,SRF seg 30 CNV (+)	31	5	4
48	TCF7L2 seg 42 CNV (+)	30	1	3
54	UBP1 seg 16 CNV (+)	18	1	0
62	APC mutation (+)	136	17	10
63	APC mutation (-)	56	3	6
65	CDNK2A mutation (-)	43	3	6
66	TP53 mutation (-)	35	11	3
67	BRAF mutation (-)	34	3	3
68	TP53 mutation (+)	33	4	6
69	CDKN2A mutation (+)	32	6	3
71	PTEN mutation (+)	20	1	2
72	KRAS mutation (-)	16	1	1
73	BRAF mutation (+)	16	3	1
75	PTEN mutation (-)	13	2	4
76	PAX8 methylation (-)	67	1	4
77	BCR methylation (-)	58	5	10
79	CCND1,PPARG methylation (-)	13	4	7
80	COL5A1 methylation (-)	10	1	1
83	TF group 1 (+)	338	236	48
				
microRNA modules				
index	drivers/regulators	N_1_	N_2_	N_3_

4	ZIC1,MITF seg 18 CNV (-)	19	3	5
5	DNMT1 seg 15 CNV (+)	15	1	2
6	SRF seg 30 CNV (+)	13	1	1
7	MAP2K4 seg 66 CNV (-)	13	2	2
10	TP53 mutation (+)	17	3	3
12	APC mutation (+)	10	1	1
14	BCR methylation (-)	12	1	4
15	SMAD3,BDNF (+)	28	8	10
20	HIF1A (+)	14	1	2

Index: module index. *N*_1_: number of passengers in the module. *N*_2_: number of co-cited driver/regulator-passenger pairs in the module. *N*_3_: max number of co-cited driver/regulator-passengers over 10 random modules of the same size. TF group 1 consists of SMAD3,FOXD1,PLAU,BDNF,FOSL2,FOSL1,RBMS. TF group 2 consists of ERG,ELK4,NFATC1,POU5F1,ZNF350.

Third, for the passenger genes in each association module we evaluated the enrichment p-values for functional categories [41] and curated pathways ([42-44]). Table 5 reports representative GO categories and pathways. 38 of the 84 association modules have at least one enriched GO category/pathway with p-value ≤ 0.001. The complete list of enriched GO categories and pathways are reported in Additional file 5, Table S4.

**Table 5:** Representative enriched GO categories and pathways in mRNA association modules

index	drivers/regulators	***N***_**1**_	function	*p*-value
44	ZIC1,MITF seg 18 CNV (+)	118	melanosome	1.300 × 10^-7^
45	TBP,MYB,HDAC2 seg 31 CNV (+)	77	RNA polymerase II regulation	1.35 × 10^-5^
45	TBP,MYB,HDAC2 seg 31 CNV (+)	77	condensed chromosome	7.928 × 10^-5^
46	SREBF2,EP300 seg 84 CNV (+)	49	cholesterol biosynthesis	3.781 × 10^-4^
47	E2F3,NFYA,SRF seg 30 CNV (+)	31	ceramide signaling pathway	4.302 × 10^-5^
47	E2F3,NFYA,SRF seg 30 CNV (+)	31	NFKB signaling pathway	8.632 × 10^-4^
48	TCF7L2 seg 42 CNV (+)	30	multi-drug resistance factors	5.884 × 10^-5^
48	TCF7L2 seg 42 CNV (+)	30	positive regulation of epithelial cell proliferation	1.096 × 10^-4^
49	NR2C1,NFYB seg 49 CNV (+)	22	mRNA translation	2.314 × 10^-4^
54	UBP1 seg 16 CNV (+)	18	RNA Pol II phosphorylation	4.429 × 10^-4^
56	APEX1 seg 57 (+)	16	cleavage of growing transcript	7.872 × 10^-4^
57	RPA2,EIF2C1 seg 16 CNV (+)	15	mRNA splicing	1.945 × 10^-6^
57	RPA2,EIF2C1 seg 16 CNV (+)	15	mRNA Pol II transcription initiation	7.260 × 10^-4^
59	RICS seg 51 CNV (+)	14	regulation of apoptosis	1.397 × 10^-4^
62	APC mutation (+)	136	liver development	4.932 × 10^-4^
66	TP53 mutation (-)	35	caspase activation	1.101 × 10^-3^
66	TP53 mutation (-)	35	P53 signaling pathway	4.138 × 10^-4^
68	TP53 mutation (+)	33	cell cycle	9.787 × 10^-4^
69	CDKN2A mutation (+)	32	cell motility	8.510 × 10^-4^
70	KRAS mutation (+)	25	DNA replication	2.689 × 10^-3^
71	PTEN mutation (+)	20	JNK cascade	2.904 × 10^-4^
75	PTEN mutation (-)	13	positive regulation of I-κB kinase/NF-κB cascade	1.745 × 10^-3^
79	CCND1,PPARG methylation (-)	13	negative regulation of cell proliferation	6.074 × 10^-3^
81	mir group 1 (-)	121	transport vesicle	3.297 × 10^-4^
82	mir group 2 (-)	121	translation	3.694 × 10^-4^
83	TF group 1 (+)	338	integrin signaling pathway	4.664 × 10^-8^
83	TF group 1 (+)	338	TGFβ signaling pathway	9.555 × 10^-4^
84	TF group 2 (+)	338	T cell differentiation	1.128 × 10^-4^

Index: module index. *N*_1_: number of passengers in the module. Function: enriched GO category or pathway. *p*-value: hyper-geometric p-value for enrichment. Mir group 1 consists of mir-92,mir-96,mir-106a,mir 20b,mir-17,mir-19b,mir-32,mir-135,mir-25,mir-106b,mir-93,mir-106,mir-18,mir-20. Mir group 2 consists of mir-24,mir-99b,mir-27b,mir-21,mir-125a,mir-23b,mir-27a,mir-23a. TF group 1 consists of SMAD3,FOXD1,PLAU,BDNF,FOSL2,FOSL1,RBMS. TF group 2 consists of ERG,ELK4,NFATC1,POU5F1,ZNF350.

#### Genes of cis-acting effects with segment CNVs exhibit a clustering tendency

We partitioned the CGH data into 86 coherent segments (Additional file 1, Text S1) and examined positive associations between segment CNVs and the mRNA expressions of their constituent genes. The cis-acting effects with segment CNVs constitute a substantial portion of the associations with mRNA expressions: 1699 of 6888 mRNA expressions can be explained by the local segment CNVs. These associations are grouped by segments into 43 modules. The members of association modules for local segment CNVs are reported in Additional file 2, Table S1, and information about partitioned segments is reported in Additional file 6, Table S5.

**Figure 2:**
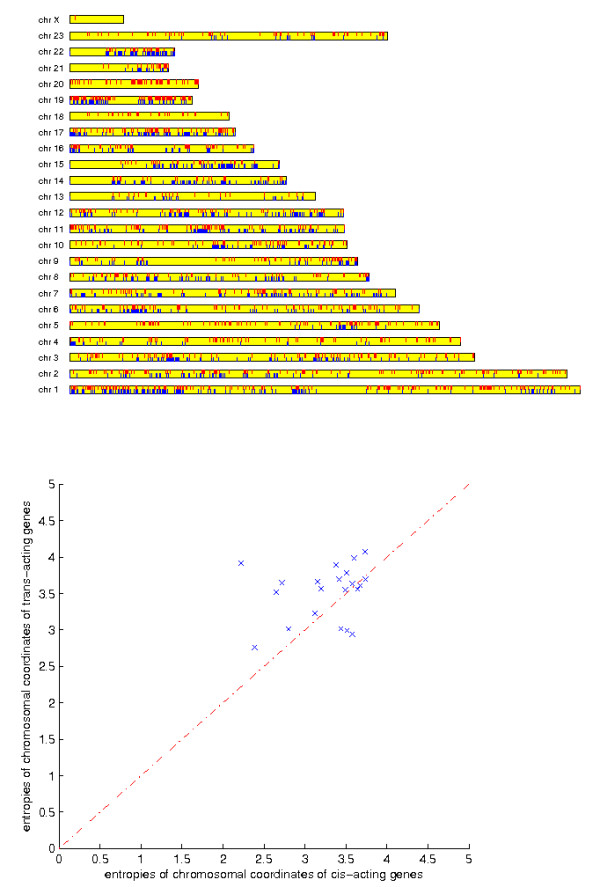
**Clustering tendency of cis-acting effects with segment CNVs**. Top: Locations of passenger genes explained by segment CNVs. Blue lines on the bottom mark the locations of passenger genes explained by cis-acting effects. Red lines on the top mark the locations of passenger genes explained by trans-acting effects. Bottom: Evenness (entropies) of the distributions of cis and trans acting genes on each chromosome. X-axis shows the entropies of cis-acting genes, Y-axis shows the entropies of trans-acting genes.

The passenger genes associated with local segment CNVs are not evenly distributed on chromosomes. Figure 2 marks the locations of these passenger genes (blue lines) on each chromosome. The passenger genes associated with local segment CNVs exhibit a clustering tendency. To rule out the possibility that the clustering patterns arise from the distributions of all protein-coding genes on chromosomes, we also marked the locations of passenger genes associated with non-local segment CNVs (trans-acting effects) in Figure 2 (red lines). Despite the presence of dense clusters, the passenger genes with trans-acting effects are more scattered on chromosomes. We evaluated the entropies (dispersions) of gene densities for cis and trans-acting genes on each chromosome (Figure 2). The trans-acting genes have higher entropies than the cis-acting genes in the majority of the chromosomes, confirming the visual observation about their differences.

#### Trans-acting effects with segment CNVs are mediated by transcription factors

**Figure 3:**
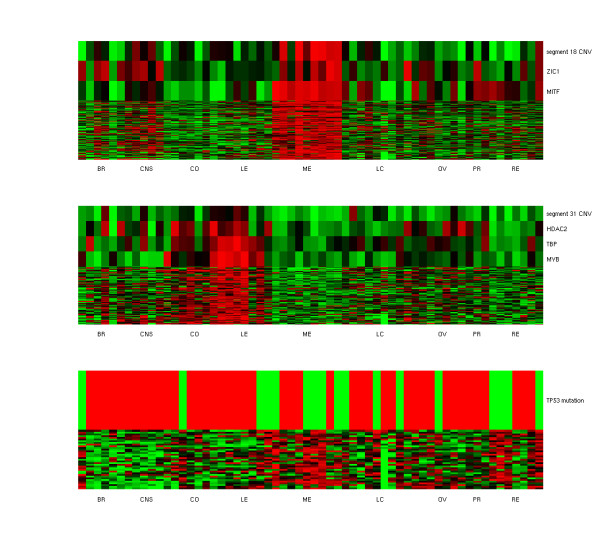
**Gene expressions of two segment CNV association modules and one mutation association module**. Top: From the top to bottom: segment 18 (chr 3p) CNV, ZIC1 expressions, MITF expressions, and expressions of passenger genes. Middle: From the top to bottom: segment 31 (chr 6q) CNV, HDAC2 expressions, TBP expressions, MYB expressions, and expressions of passenger genes. Bottom: From the top to bottom: TP53 mutation states and expressions of passenger genes. Passengers are ordered by their similarity to driver aberration profiles. The top row has the highest similarity. BR: breast cancers, CNS: brain tumors, CO: colorectal cancers, LE: leukemias, ME: melanomas, LC: lung cancers, OV: ovarian cancers, PR: prostate cancers, RE: renal cancers.

506 mRNA expressions are associated with non-local segment CNVs. One possible mechanism to establish these trans-acting associations is through intermediate regulators on segments: segment CNVs have cis-acting effects on the expressions of their constituent regulators, which in turn modulate the expressions of downstream targets on other loci. We identified 18 association modules with intermediate regulators and reported them in Table 1. Module 44 has segment 18 (chromosome 3p) as the driver, MITF and ZIC1 as intermediate regulators, and 118 passengers. The driver CNV as well as regulator and passenger mRNAs all exhibit melanoma-specific elevation in NCI-60 cell lines (Figure 3). MITF encodes a transcription factor that regulates the differentiation and development of melanocytes and pigment cell-specific transcription of the melanogenesis enzyme genes. ZIC1 encodes a zinc finger transcription factor involved in brain development. Multiple lines of evidence support the associations with MITF in this module. First, amplification of the chromosome 3p region covering MITF is considered as a typical driver mutation in melanoma [26]. Second, the passenger genes in this module are enriched with experimentally validated MITF targets from [45] (14 of 118 genes, hyper-geometric p-value ≤ 1.036 × 10^-13^), and with genes belonging to the GO category of melanosome (4 of 7 genes in the GO category, hyper-geometric p-value ≤ 1.3 × 10^-7^). Third, promoters of passenger genes are also enriched with MITF-binding motifs (Table 3, 23 of 118 passenger genes containing the motif, hyper-geometric p-value ≤ 0.0464). Fourth, MITF and 12 of the 118 passenger genes are co-cited in previous publications (Table 4).

Module 45 has segment 31 (chromosome 6q) as the driver, MYB, TBP and HDAC2 as intermediate regulators, and 77 passengers. The driver CNV as well as regulator and passenger mRNAs all exhibit leukemia-specific elevation (Figure 3). MYB (c-myb) is a transcription factor involved in cell cycle progression, cell proliferation and differentiation in hematopoiesis. Amplifications of this oncogene cause its abnormal expressions in leukemia and other solid tumors [46,47]. TBP is a TATA-binding protein belonging to the general transcription apparatus. HDAC2 is a histone deacetylase that modifies chromatin structures and represses transcription. Associations of MYB and TBP with the passenger genes are supported by enrichment of their binding motifs on promoters (Table 3, 65 of 77 genes for MYB motifs, hyper-geometric p-value ≤ 0.0076, 56 of 77 genes for TBP motifs, hyper-geometric p-value ≤ 5.370 × 10^-4^) and co-citations of regulators and passengers in literature (Additional file 4, Table S3, 14 passenger genes are co-cited with MYB, and 8 passenger genes are co-cited with TBP). Intriguingly, the passenger genes in this module are highly enriched with the GO categories or pathways involved in generic DNA and RNA synthesis such as RNA Pol II regulation and chromatin remodeling (Table 5). The elevated activities of DNA and RNA synthesis probably reflect high division rates of leukemia: leukemia has the second lowest (next to colon cancers) average doubling time among the NCI-60 cell lines [48].

**Figure 4:**
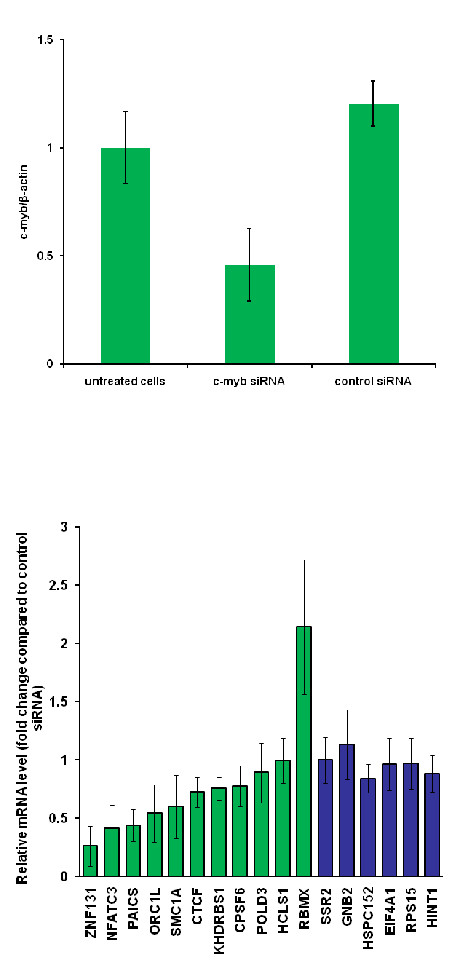
**Validation of putative MYB targets with siRNA treatments**. Top: qRT-PCR measurements of c-myb mRNA levels in the K562 leukemia cell line without treatment (left), treated with the siRNA against c-myb (middle), and treated with the siRNA against GFP (right). Bottom: Relative mRNA levels of selected genes under the c-myb siRNA treatment with respect to the qRT-PCR measurements under the control siRNA treatment. Green bars: expression responses of putative MYB targets. Blue bars: expression responses of control genes.

Associations do not necessarily imply causality as multiple types of causal structures may yield the same statistical associations [29]. We confirmed the causal relations of MYB and its associated genes by intervention experiments. We selected 11 putative target genes whose mRNAs were associated with segment 31 CNV and MYB expressions in multiple datasets (see Materials and Methods and Additional file 1, Text S1). As a control we selected 6 additional genes with high expression levels across all 60 cell lines. The K562 leukemia cell line was treated with c-myb siRNA, and the expression responses of putative MYB targets and control genes were measured by qPCR. Expressions of 6 of 11 putative MYB targets were down-regulated with the t-test p-value ≤ 0.05 under the c-myb siRNA treatment: CTCF, KHDRBS1, NFATC3, ORC1L, PAICS, and ZNF131, and RBMX was significantly up-regulated (Figure 4, green bars; Additional file 7, Table S6). In contrast, the control genes were not differentially expressed under the c-myb siRNA treatment (Figure 4, blue bars; Additional file 7, Table S6). The primers and the probe used for detecting each gene are listed in Additional file 8, Table S7.

Module 46 has segment 84 (chromosome 22q) as the driver, SREBF2 and EP300 as intermediate regulators, and 49 passengers. SREBF2 encodes a sterol regulatory element binding transcription factor. The SREBF2-binding motif is not enriched on the passenger promoters (hyper-geometric p-value ≤ 0.1581, Table 3). However, two passenger genes are involved in cholesterol biosynthesis (FDFT1 and HMGCR), and the enrichment is significant (p-value ≤ 3.781 × 10^-4^, Table 5).

Module 47 has segment 30 (chromosome 6p) as the driver, E2F3, NFYA and SRF as intermediate regulators, and 31 passengers. The passenger genes are moderately enriched with E2F3-binding motifs (Table 3, 21 of 31 genes, p-value ≤ 0.0989), and five (regulator, passenger) pairs are co-cited in the same studies (Table 4). SRF (serum response factor) is known to regulate RAF1 (serine/threonine-protein kinase), a member of passenger genes [49]. Moreover, SRF and RAF1 co-participate in multiple signaling pathways including IGF1, PDGF, and MAPK pathways (Additional file 5, Table S4). SRF is also associated with two other passenger genes (RELA and SMAD4) in previous studies (Additional file 4, Table S3).

#### Associations with aberrations on individual genes or microRNAs reveal the causal relations of their targets

Associations with aberrations on individual genes or microRNAs provide direct causal explanations for gene expressions. 894 mRNA expressions are associated with aberrations on individual genes or microRNAs - mutations, DNA methylations of genes and microRNA expressions. They are grouped into 21 modules and are reported in Table 1. The causal relations between drivers and passengers are supported by previous studies in 20 association modules.

Positive and negative associations with gene mutations comprise 12 mRNA expression modules and 522 passenger genes. APC encodes a tumor suppressor protein that acts as an antagonist of the Wnt signaling pathway and is involved in other processes such as cell migration and adhesion. APC is mutated exclusively in colon cancer cell lines. APC mutations are positively associated with 136 passenger genes and negatively associated with 56 passenger genes (Additional file 1, Figure S9). More previous studies support the positive associations with APC mutations: 21 passenger genes are co-cited with APC in previous publications. Some of these passenger genes are members or downstream targets of the Wnt signaling pathway: BMP4, CD9, CDH1, GPX2, HDAC1, PPARG, and SOX9 (Additional file 4, Table S3). Mutations of APC activate the Wnt pathway thus up-regulate its target genes. Other passenger genes are associated with APC aberrations in selected tissue or disease samples: DDR1, DLG3, ETS2, HOXA10, ITGB4, MEST, PLA2G4B, SIGIRR, and SPINT2 (Additional file 4, Table S3). In contrast, only 4 negative associations with APC are supported by prior publications. APC and PSMD2 are in the pathway of β-catenin degradation (Additional file 5, Table S4). APC and CMTM3, EXO1 and HLTF are reported to be hyper-methylated in selected colon cancer samples (Additional file 4, Table S3).

CDKN2A (TP16) encodes a tumor suppressor that inhibits cyclin-dependent kinase 4 (CDK4) in cell cycle control. It possesses loss-of-function mutations (mostly frameshift insertions/deletions) in 33 samples. CDKN2A mutations are negatively associated with 43 passenger genes and positively associated with 32 passenger genes (Additional file 1, Figure S10). Three negative associations are supported by previous studies: ERBB3, FOS and HSD17B4 (Additional file 4, Table S3). Six positive associations are supported by previous studies: CAV1, LIMK1, MSN, THBD, TRDMT1, and YWHAG (Additional file 4, Table S3). Moreover, the positively associated genes are enriched with the GO category of cell motility (p-value ≤ 8.51 × 10^-4^) and signaling pathways of cell motility (p-value ≤ 2.28 × 10^-3^) and integrin (p-value ≤ 2.42 × 10^-3^).

TP53 encodes a master regulator for apoptosis, cell cycle control and senescence in response to stress conditions. TP53 mutations are negatively associated with 35 passenger genes and positively associated with 33 passenger genes (Additional file 1, Figure S9). Negative associations are highly enriched with known TP53 targets from multiple lines of evidence. First, the passenger genes are enriched with a list of 45 known TP53-responsive genes confirmed by ChIP-Seq assays ([50], 7 of 35 genes, hyper-geometric p-value ≤ 7.6608 × 10^-12^). Second, promoters of the passenger genes are enriched with TP53-binding motifs (16 of 35 genes, hyper-geometric p-value ≤ 0.0041, Table 3). Third, 11 of the 35 passenger genes are co-cited with TP53 in previous publications (Table 4): BAX, CCNG1, CDKN1A, DDB2, EGFL7, FDXR, GDF15, LTBR, MDM2, TNFRSF10B (Additional file 4, Table S3). Most of these co-citations pertain to well-known pathways of TP53 regulation. For instance, MDM2 is a ubiquitin ligase and transcriptional target of TP53, CDKN1A (TP21) is a cyclin dependent kinase inhibitor and transcriptional target of TP53, BAX is a BCL2-associated protein involved in apoptosis. Fourth, the passenger genes are enriched with GO categories of caspace activation (hyper-geometric p-value ≤ 0.001), cyclin-dependent kinase activity (p-value ≤ 0.0025), cell cycle arrest (p-value ≤ 0.0069), and pathways of TP53 signaling (p-values ≤ 1.55 × 10^-5^,≤ 4.14 × 10^-4^), and G2/M checkpoint (p-value ≤ 0.0012) (Table 5). In contrast, positive associations with TP53 mutations are not enriched with known TP53 targets or promoters containing TP53-binding motifs. Negative associations with TP53 mutations imply positive regulation of TP53 since most TP53 mutations are loss-of-function mutations.

161 mRNA expressions are negatively associated with DNA methylations of five gene clusters (Table 1). They include the association modules of PAX8 (61 passenger genes), BCR (58 passenger genes), HOXC13 (19 passenger genes), CCND1 and PPARG (13 passenger genes), and COL5A1 (10 passenger genes). The passenger genes of PAX8 and HOXC13 modules are enriched with the binding motifs of their drivers (PAX8: 34 of 61 genes, hyper-geometric p-value ≤ 0.0014, HOXC13: 12 of 19 genes, hyper-geometric p-value ≤ 0.016). Furthermore, a passenger gene in the CCND1/PPARG module, KLF4, is regulated by both CCND1 and PPARG from multiple previous studies (Additional file 4, Table S3).

241 mRNA expressions are negatively associated with two clusters of microRNA expressions (Table 1). Association module 81 possesses mir-92, mir-96, mir-106a, mir-20b, mir-17, mir-19b, mir-32, mir-135, mir-25, mir-106b, mir-93, mir-106, mir-18, and mir-20 as drivers and 121 passenger genes. Association module 82 contains mir-24, mir-99b, mir-27b, mir-21, mir-125a, mir-23a, mir-23b, and mir-27a as drivers and 120 passenger genes. The passenger genes in both modules are enriched with the predicted targets of their driver microRNAs (Table 3, association module 81: mir-96, p-value ≤ 0.015, mir-106a, p-value ≤ 0.046, mir-17, p-value ≤ 0.038, mir-93, p-value ≤ 0.032; association module 82: mir-21, p-value ≤ 0.001). Therefore, some passenger mRNA expressions in these modules are probably regulated by their driver microRNAs.

#### Gene clusters with coherent expression profiles are likely to be regulated by transcription factors

**Figure 5:**
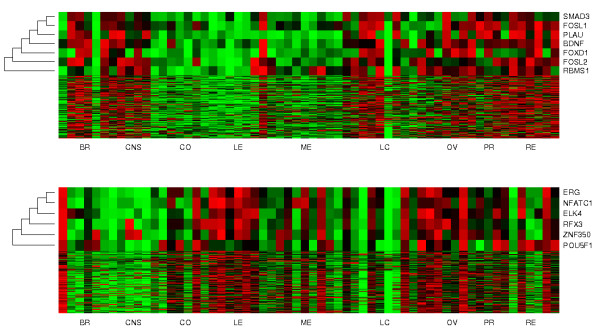
**Gene expressions of two TF expression association modules**. Top: From the top to bottom: expressions of putative regulators of the module (SMAD3, FOXD1, PLAU, BDNF, FOSL2, FOSL1, RBMS1) and expressions of passenger genes. Bottom: From the top to bottom: expressions of putative regulators of the module (ERG, ELK4, NFATC1, RFX3, POU5F1, ZNF350) and expressions of passenger genes. Regulators are ordered by hierarchical clustering with their aberration profiles, and their dendrogram are displayed on the left of the panel. Passengers are sorted by their average similarity to the driver aberration profile. The top row has the highest similarity. BR: breast cancers, CNS: brain tumors, CO: colorectal cancers, LE: leukemias, ME: melanomas, LC: lung cancers, OV: ovarian cancers, PR: prostate cancers, RE: renal cancers. Samples 1 and 5 are estrogen-positive breast cancers, and samples 2-4 are estrogen-negative breast cancers.

A unique characteristic of NCI-60 mRNA expression data is the presence of a few large clusters with highly coherent expression profiles (Additional file 1, Figures S1, S2, S3, S4, S5, S6, S7, S8, S9, S10, S11, S12, S13). Two of the largest clusters are not associated with any observed molecular aberrations. We identified the transcription factors that were associated with the mRNA expressions in these clusters as candidates for intermediate regulators. These transcription factors may regulate the coherent expressions of downstream genes, yet the drivers that control the regulator expressions are unknown. Two association modules with intermediate regulators are reported in Table 1. Module 83 contains SMAD3, FOXD1, PLAU, BDNF, FOSL2, FOSL1 and RBMS1 as regulators and 338 passenger genes. Module 84 contains ERG, ELK4, NFATC1, RFX3, POU5F1 and ZNF350 as regulators and 338 passenger genes. The expression profiles of the two modules are shown in Figure 5.

Associations of regulators and passengers in these modules are supported by rich prior studies. Promoters of module 83 passengers are highly enriched with SMAD3-binding motifs (Table 3, 271 of 338 genes, hyper-geometric p-value ≤ 1.058 × 10^-7^). SMAD3 encodes a transcriptional modulator activated by transforming growth factor-*β *(TGF*β*). Indeed, many passenger genes are members or downstream targets of the TGF*β *signaling pathway according to previous studies, such as TGFBR2, ADAM12, CAV1, CCNB1, and EGFR (Table 6 and Additional file 4, Table S3). In addition, many passenger genes are co-cited with other candidate regulators (PLAU, BDNF, FOSL1) in previous publications. For instance, associations of BDNF and passenger genes such as ADAM17, AXL, and BCAT1 in neuron cells are previously reported (Additional file 4, Table S3).

**Table 6:** Summary information of association modules for microRNA expressions

index	driver type	drivers	reg	sign	N
1	inter CNV	seg 69	SPOP,NME2,POLG2	-	38
2	inter CNV	seg 55	NR2C1,NFYB	-	29
3	inter CNV	seg 51	RICS	+	23
4	inter CNV	seg 18	ZIC1,MITF	-	19
5	inter CNV	seg 15	DNMT1,MBD3	+	15
6	inter CNV	seg 30	SRF	+	13
7	inter CNV	seg 66	MAP2K4	-	13
8	inter CNV	seg 68	BRCA1	-	12
9	inter CNV	seg 13	NFE2L2	-	10
10	mutation	TP53	NA	+	17
11	mutation	KRAS	NA	-	15
12	mutation	APC	NA	+	10
13	mutation	STK11	NA	-	10
14	methylation	BCR	NA	-	12
15	TF	TF group	1 NA	+	28
16	TF	MBD1	NA	+	19
17	TF	TFE3	NA	+	18
18	TF	BR2F1	NA	+	17
19	TF	GRLF1	NA	+	15
20	TF	HIF1A	NA	+	14
21	TF	E4F1	NA	+	12
22	TF	TFDP1	NA	+	12
23	TF	FOXM1	NA	+	11
24	TF	ATF2	NA	+	10
25	intra CNV	chr14p	NA	+	14

Annotations follow Table 1.

Promoters of module 84 passengers are enriched with the binding motifs of NFATC1 (265 of 388 genes, hyper-geometric p-value ≤ 3.15 × 10^-5^), POU5F (18 of 338 genes, hyper-geometric p-value ≤ 0.0027), ERG (67 of 338 genes, hyper-geometric p-value ≤ 0.0093), RFX3 (75 of 338 genes, hyper-geometric p-value ≤ 0.0481), and ELK4 (76 of 338 genes, hyper-geometric p-value ≤ 0.0518). Many passenger genes are co-cited with some of these regulators in previous studies (Table 4).

#### Most association modules exhibit tissue-specific expressions

**Figure 6:**
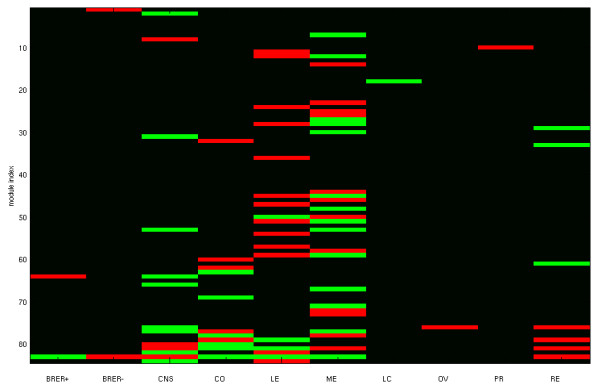
**Tissue-specific patterns of mRNA association modules**. Each row represents the tissue-specific pattern of a module. Red: coherent up-regulation in a tissue type. Green: coherent down-regulation in a tissue type. Black: incoherent expressions in a tissue type. Cis-acting modules range from 1 to 43. Trans-acting modules range from 44 to 84. BRER+: ER-positive breast cancers, BRER-: ER-negative breast cancers, CNS: brain tumors, CO: colorectal cancers, LE: leukemias, ME: melanomas, LC: lung cancers, OV: ovarian cancers, PR: prostate cancers, RE: renal cancers.

NCI-60 cell lines constitute 9 distinct tissue origins. The patterns of molecular aberrations and gene expressions are likely to vary between tissue types. The inferred modules capture strong associations between drivers and passengers across all samples. Strong associations, however, can be manifested by either tissue-specific or tissue-independent patterns. These two types of patterns are illustrated in Figure 3. The driver of module 44 (segment 18 CNV) is amplified in melanoma cell lines alone, and its passenger expressions are up-regulated in melanoma cell lines. In contrast, the driver of module 66 (TP53 sequence) has prevalent mutations among all tissue types, and its passenger expression patterns are independent of tissue types. To check whether the effects of dysregulation are universal or tissue-specific, we extracted the tissue-specific patterns of drivers and passengers in each module (see descriptions in Materials and Methods and Additional file 1, Text S1) and visualized them in Figure 6. We observed the following intriguing results. First, about half of the cis-acting modules (internal segment CNVs as drivers) are coherently up or down regulated in at least one tissue type. In contrast, the majority of the trans-acting modules (all other types of drivers) have the same property. Second, despite many association modules possess tissue-specific patterns, none of them are coherently up or down regulated among all tissue types. In other words, a module has incoherent driver aberrations and passenger expressions in at least one tissue type. Third, the tissue-specific patterns are dominated by four tissue types: melanoma, leukemia, colon and central nervous system. For instance, 8 and 4 of 41 trans-acting modules contain up and down regulated passengers in leukemia respectively. 8 and 9 of 41 trans-acting modules contain up and down regulated passengers in melanoma respectively. 5 trans-acting modules contain differentially expressed passengers in renal cancers. In contrast, very few modules possess tissue-specific patterns on lung, ovarian or prostate cancers. Fourth, no module is coherently up or down regulated in breast cancer cell lines. Yet several of them possess coherent expressions in estrogen (ER)-positive or ER-negative samples. For instance, module 83 (Figure 5) passengers are up-regulated in ER-negative samples and down-regulated in ER-positive samples. Fifth, tissue-independent patterns belong to transacting modules with external segment CNVs or mutations as drivers. Five of 14 modules associated with mutations are tissue-independent: module 65 (CDKN2A), module 68 (TP53), module 70 (KRAS), module 74 (PIK3CA) and module 75 (PTEN). The mutational states of these genes on the samples are not aligned with their tissue types. In contrast, modules associated with mutations of APC and BRAF possess tissue-specific patterns, as APC and BRAF mutations occur primarily on colon cancer and melanoma samples respectively. Similarly, the majority of microRNA association modules possess tissue-specific patterns, and these patterns are dominated by up/down regulations in leukemias, central nervous systems and colons. Different from mRNA modules, only two microRNA modules possess melanoma-specific patterns. The tissue-specific patterns of microRNA modules are displayed in Additional file 1, Figure S17.

### MicroRNA expressions are driven by distinct aberration mechanisms from mRNA expressions

195 of 303 microRNA expressions are associated with observed molecular aberrations. We grouped these associations into 25 modules and summarized their information in Table 6. Intriguingly, the drivers of the top-ranking microRNA modules are marginally overlapped with those of the top-ranking mRNA modules, suggesting that microRNA and mRNA expressions are driven by distinct molecular aberrations. The complete list of association modules are reported in Additional file 3, Table S2, and driver aberrations and passenger microRNA expressions are visualized in Additional file 1, Figures S14, S15, S16.

Table 2 shows the FDRs for each type of associations and all association together for microRNA modules. The microRNA FDRs exhibit same trend as the mRNA FDRs. Associations with segment CNVs yield the lowest rates, followed by associations with mutations and transcription factor expressions. Associations with DNA methylation yield the worst performance. Associations with microRNA expressions and local segment CNVs were not considered. The overall FDRs calculated by the two methods are 0.292 and 0.394 respectively.

Information about regulators and functions of microRNAs is much more scattered than those of protein-coding genes. We incurred a batch search on PubMed to identify co-citations of drivers/regulators and passenger microRNAs in these modules. Overall, co-citations of drivers/regulators and passengers were found in 9 associations modules. Table 4 and Additional file 4, Table S3 report the co-citation outcomes for microRNA modules.

#### Cis-acting effects with segment CNVs are absent on microRNAs

A remarkable distinction between mi-croRNA and mRNA associations is the lack of associations with local segment CNVs in microRNA modules. Mi-croRNA expressions are poorly correlated with their local segment CNVs compared to mRNA expressions (Additional file 1, Figure S18). The only possible exception is a module of microRNAs located on chromosome 14p (Additional file 3, Table S2). These microRNAs exhibit a coherent expression pattern (Additional file 1, Figure S16) and are localized on chromosome 14p. Thus they are likely to be modulated by local segment CNVs. Yet we cannot verify these associations since chromosome 14p is not covered by the CNV data.

#### Associations suggest the existence of feedback loops between microRNAs and protein-coding genes

Co-citation search outcomes demonstrate bidirectional regulatory links between the driver/regulator genes and passenger mircroRNAs. Table 7 summarizes these regulatory links reported from prior studies. Most drivers/regulators of these microRNA modules are known or predicted targets of the passenger microRNAs. Hence there are possible bidirectional links connecting transcription factors and microRNAs. In some cases, bidirectional links are reported. For instance, MAP2K4 in module 7 is both a known regulator and target of two passenger microRNAs mir-25 and mir-92. Similarly, TP53 in module 10 is both a known regulator and target of two passenger microRNAs mir-15a and mir-16.

**Table 7:** Regulatory links between drivers/regulators and microRNAs

index	link	PMID	index	link	PMID
4	mir-182, mir-183 →MITF	17597072	15	mir-21 → SMAD3	19816956
4	mir-130b → ZIC1	20676061	15	mir-23a → SMAD3	18508316
5	DNMT1 → let-7a	17308078	15	mir-24 → SMAD3	18353861
6	SRF → mir-143	21069820	15	mir-10a → BDNF	20309390
7	mir-25 ↔ MAP2K4	19861690, 15652477	15	mir-125b → BDNF	19635812
7	mir-92 ↔ MAP2K4	17683260, 15652477	15	mir-134 → BDNF	20622856, 16421561
10	mir-15a, mir-16 ↔ TP53	19347736, 15652477	15	mir-210 → BDNF	19826008
14	mir-203 → BCR	18538733	20	mir-34c → HIF1A	20861672

Index: microRNA association module index. Link: regulatory links between regulators and microRNAs. Arrows indicate the directions of regulation. PMID: the PubMed ID of the reported link.

## Discussion

Uncovering causal relations of genes from static, observational data alone is challenging due to the ambiguities of transferring associations into causal information. We address this problem by exploiting two widely accepted hypotheses in cancer genomics: (1)molecular aberrations on DNAs (mutations, copy number variations, DNA methylations) affect the levels of RNA transcripts but not the other way around, (2)if both cis-acting and trans-acting effects of segment CNVs are detected, then cis-acting effects are more likely to be the direct causes for expression changes. Based on these hypotheses we built association modules between putative driver molecular aberrations and passenger mRNA or microRNA gene expressions. Our predictions are supported by various validation results. First, the downstream targets of several well-known cancer-related genes such as MITF, TP53 and APC are retrieved from our association modules. Second, the passenger genes of large modules are enriched with the putative targets of their regulators or drivers. Third, putative targets of MYB are down-regulated under the MYB siRNA treatment. Fourth, associations between molecular aberrations and microRNA expressions reveal the bidirectional regulatory links between transcription factors and microRNAs.

One intriguing observation is that each type of molecular aberrations participate in at least one association module. Therefore, to track the causes of expression variations it is necessary to interrogate multiple types of molecular aberrations concurrently. NCI-60 datasets are sparse in mutations (24 valid genes), copy number variations (219 valid genes) and DNA methylations (320 valid genes). Consequently, about half of the mRNA expression data (3571 genes) are not significantly associated with observed aberrations. This drawback will become less prominent in new generations of comprehensive cancer genomics projects (e.g., TCGA [7] and ICGC [8]), as they employ high density microarrays and next-generation sequencing to cover the entire genomes.

Associations with driver molecular aberrations can be extended to clinically relevant phenotypes such as drug resistance [51]. Unlike mRNA or microRNA expressions, these phenotypes are not the direct products of molecular aberrations. Thus associations are likely to be mediated by the genes functionally related to the phenotypes. For instance, from the drug response data on NCI-60 cell lines [19] we found leukemia cell lines were sensitive to the majority of compounds tested (data not shown). This observation coincides with up-regulation of genes involved in transcriptional and translational processes in leukemia (Additional file 5, Table S4). Both drug responses and gene expressions reflect the high growth and division rates of leukemia cell lines relative to other samples.

Two of the largest association modules (modules 83 and 84) lack driver aberrations but consist of intermediate regulators. In particular, multiple lines of evidence indicate the expressions of module 83 are controlled by TGFβ pathway activities. Yet it is unclear which molecular aberrations modulate the expressions of the intermediate regulators. Examination of the mutations and DNA methylations of members on the TGFβ pathway may reveal their driver aberrations.

Cis-acting and trans-acting effects with segment CNVs dominate the association modules of mRNA expressions (61 of 84 association modules). Amplifications and deletions of chromosomal segments are powerful driver aberrations, as they can possibly affect many genes on the segments and downstream targets on other loci. Determination of cis-acting and trans-acting effects with CNVs remains an open problem. We observed a tendency of cis-acting genes to cluster on certain hot-regions of chromosomes. Yet many cis-acting genes are still scattered around the entire chromosomes. The distributions of cis and trans-acting genes will become more clear from the data of high density CGH arrays.

Associations with mutations of several cancer-related genes - TP53, CDKN2A, APC - are largely consistent with their known targets. However, the passenger sets of positive and negative associations exhibit differential levels of enrichment. Passenger genes negatively associated with TP53 mutations (module 66) are enriched with TP53-binding motifs (Table 3) and known targets of TP53 (Table 5 and [50]). In contrast, passenger genes positively associated with TP53 mutations (module 68) do not possess the enrichment. TP53 undergoes loss-of-function mutations, thus negative associations imply positive regulation between TP53 and its targets. Although negative regulation of TP53 has been previously reported [52], on NCI-60 TP53 seems to be an activator of its prominent direct targets. The enriched functional information is reversed in APC and CDKN2A mutations. 15 of the passenger genes positively associated with APC mutations (module 62) are involved in the Wnt pathway or associated with APC. In contrast, only 3 of the passenger genes negative associated with APC mutations (module 63) are related to APC. Similarly, 5 of the passenger genes positively associated with CDKN2A mutations (module 69) are related to CDKN2A, whereas only 2 of the passenger genes negatively associated with CDKN2A mutations (module 65) are related to CDKN2A. Unlike TP53, APC and CDKN2A are inhibitors of signaling pathways (Wnt and cell cycle control) and possess no direct targets. Hence their loss-of-function mutations would activate the downstream targets of the pathways. In contrast, genes down-regulated in APC or CDKN2A mutants are likely to arise from secondary effects instead of direct consequences of APC or CDKN2A mutations.

Two large clusters of mRNA expressions are negatively associated with groups of microRNA expressions (modules 81 and 82). The causality between microRNAs and mRNAs are ambiguous since regulation can take place in both directions. The passenger genes are enriched with the predicted targets of driver microRNAs. Additional evidence is required to determine their causal directions.

In NCI-60 cell lines, microRNA and mRNA expressions seem to be driven by distinct molecular aberrations. Unlike mRNA expression modules, none of the microRNA expression modules are associated with local segment CNVs. This is curious since many microRNAs in this study are located on the partitioned segments and between the protein-coding genes possessing the cis-acting effects with segment CNVs. Poor correlations between microRNA expressions and their local segment CNVs suggest either the low resolution of the CGH data or the trans-acting nature of microRNA regulation.

It is more difficult to validate the association modules of microRNA expressions due to the paucity of regulatory and functional information. Intriguingly, we found that many drivers and regulators were known or predicted targets of their passenger microRNAs (Table 7). In some cases, regulation along both directions between drivers and passengers is reported (for instance, MAP2K4 v.s. mir-25 and mir-92). The association outcomes and prior studies suggest feedback regulation between driver transcription factors and passenger microRNAs.

It is worth noting that pairwise associations inferred from the data are projections of multi-variate models from drivers to the target passenger. A driver links to a passenger if their association cannot be explained by other driver variables. Therefore, "pairwise associations" in this study carry a very different meaning from purely pairwise scores such as correlation coefficients. A more adequate analogy is conditional independence in probabilistic graphical models. A driver A links to a passenger X if A is not conditionally independent of X given any other driver variable. Such direct and indirect relations can be captured by a variety of prior approaches such as Bayesian networks, mutual information and regression. Our contribution is not to invent/introduce a new mathematical tool to tackle the same problem, but to develop a data integration framework to exploit the statistical and mechanistic properties of the data. More specifically, the novelty of this work includes (1)Prioritize the order of adding association links according to mechanistic information. Cis-acting effects are first considered as they provide direct explanation for passenger expression fluctuations. Trans-acting effects with aberrations on DNA as drivers (CNV, mutations, DNA methylations) are taken into account only if they provide additional explanatory power relative to cis-acting effects. Finally, trans-acting effects without DNA aberrations (microRNA or transcription factor expressions) are introduced on top of the lower layered models. (2)Project the complex network of conditional independence relations onto pairwise links and group these links as driver-centric association modules. This simplification is useful and practical as it is much easier to examine and validate a coherent module than a complex network.

Projecting a complex regulatory network onto well-structured modules certainly yields information loss. Genes are often regulated by combinatorial interactions of multiple transcription factors, chromatin modifiers and signaling proteins. Moreover, the regulatory programs may vary with tissue types. In this work, we chose to ignore combinatorial interactions and tissue-specific regulatory programs and focused on strong marginal effects manifested on the variations across all 60 cell lines. This simplification seems necessary given the limited size of NCI-60 data. A new generation of cancer genomic data - such as TCGA and ICGC - often cover much more patients with a specific tumor type. Therefore, it is possible to include the combinatorial interactions and tissue-specific regulations in an extension of the current models.

The FDR values clearly depend on the constraints of associations. Internal segment CNVs require the physical proximity of drivers and passengers, and external segment CNVs require the existence of intermediate regulators linking segment CNVs and passenger expressions. These additional constraints push the FDRs below significant levels. In contrast, modules of mutations, DNA methylations, microRNA and TF expressions as drivers rely only on associations between drivers and passengers. Thus their FDRs are substantially higher than standard significant values. To lower the FDRs on those modules, additional constraints such as evidence of physical interactions between drivers and passengers are probably needed. However, since the FDRs reported in Table 2 are the aggregate results over all modules of the same type, the high values do not deteriorate the relevance of individual modules. For instance, multiple lines of evidence support the modules associated with TP53 mutations, CDKN2A mutations, PAX8 methylations, as well as TF groups 1 and 2 expressions. It is difficult to discriminate these biologically relevant modules from potentially spurious modules from the NCI-60 data alone. External information or experimental validation has to be incorporated. In this work, we use external information and experimental validation to justify the models inferred from data alone. A refined version of the model construction algorithm should include these additional information in the loop.

Only 5 transcription factor binding motifs are significantly enriched on module passengers after Bonferroni correction (TBP, PAX8, SMAD3, NFATC1 and POU5F). These unsatisfactory results can be attributed to both the conservative nature of Bonferroni correction and the accuracy of motif presence as a proxy for regulation. We sought the presence of motifs within 5kb upstreams of the transcription start sites. This simple scheme may create many false positives and false negatives. For instance, only 27 of the 113 experimentally validated MITF targets [45] contain the MITF-binding motif within 5kb promoters, and only 13 of the 67 experimentally validated TP53 targets [50] contain the TP53-binding motif within 5kb promoters. The discrepancy between motif presence and experimentally validated targets may partially explain the relatively high motif enrichment p-values of modules 44 (MITF) and 66 (TP53). Restrictions to promoters with multiple motif occurrences reduce false positives but increase false negatives. The enrichment results are worse than Table 3 (Additional file 9, Table S8). Consequently, we should treat motif enrichment analysis as one error-prone validation and use it together with other validations (co-citations, functional category or pathway enrichments). Other types of validations are also subjected to error. It remains an open problem to systematically validate the large-scale models inferred from high-throughput data.

In this work, we built association modules from NCI-60 data alone and attempted to validate the inferred modules with external information. An alternative and common approach is to start with prior models using external information (e.g., pathways, GO categories, protein-DNA and protein-protein interactions) and learn the models explained by the data. As a simple comparison, we treated each pathway and GO category as the passenger gene set of a module and identified the drivers that significantly explained their expressions. The results are reported in Additional file 10, Table S9. Overall, only a small number of GO categories and pathways are significantly explained by driver aberrations in NCI-60 data. Prominent ones include genes involved in melanosome and melanin biosynthesis and explained by MITF transcription factor expressions, ribosome genes explained by a cluster of 9 microRNAs, and members of proteasome genes explained by internal segment CNVs on chromosome 14q. Notice that genes involved in melanosome and melanin biosynthesis substantially overlap with module 44.

## Conclusions

We proposed a computational method to build associations between molecular aberrations (drivers) and mRNA/microRNA expressions (passengers) and grouped the genes/microRNAs into modules according to their associated molecular aberrations. Intriguingly, a considerable portion of these associations implied causal relations according to in-silico and experimental validations. We found passengers in these association modules were enriched with the putative targets of the drivers, functional categories and pathways in which the drivers were involved, and regulatory/association links confirmed by prior publications. Moreover, we demonstrated that the predicted MYB targets were down-regulated in a MYB-siRNA treated leukemia cell line. The reported association modules indicated that gene/microRNA expressions in cancer were driven by diverse aberration mechanisms including copy number variations, mutations, DNA methylations, microRNA and transcription factor expressions. In addition, the results suggested distinct driver mechanisms between mRNA and microRNA expressions and the existence of bidirectional regulatory links between microRNAs and transcription factors.

## Methods

### Data sources and processing

NCI-60 constitutes a panel of 60 cell lines derived from 9 tissue types: breast (5 cell lines), central nervous system (6 cell lines), colon (7 cell lines), leukemia (6 cell lines), melanoma (10 cell lines), lung (9 cell lines), ovary (7 cell lines), prostate (2 cell lines) and kidney (8 cell lines). Melanoma cell line MDA-N is derived from MDA-MB435, thus the two cell lines are highly similar. Among the 5 breast cancer cell lines 2 are estrogen-positive (MCF7 and T47D) and the remaining 3 are estrogen-negative (MDA-MB-231, HS578T. BT-549).

Seven datasets of NCI-60 cell lines were downloaded from the website of the Genomics and Bioinformatics Group at NCI (GBC): mutation analysis of 24 cancer genes [10], Comparative Genomic Hybridization (CGH) array data of DNA copy number variations [11], cytosine methylation profiling on promoters [12], cDNA microarray data [15], Affymetrix transcript profile data [16], and Agilent transcript profile data [17] of mRNA expressions, and microRNA expression data [13]. The union of these datasets covered 14856 genes and 303 microRNAs.

Continuous data (CNVs, mRNA expressions, microRNA expressions, DNA methylations) were first rank-transformed into cumulative distribution function (CDF) values and then converted into probability vectors of trinary states using probabilistic quantization [33]. This transformation normalizes the data in the same scale and preserves the information from continuous data. Discrete data (mutations) was directly fed into the model without processing. Detailed procedures of data normalization and combination are reported in Additional file 1, Text S1.

Spatial dependency of CNV data was manifested from the measurements of 219 genes in a CGH array [33]. Adjacent probes on the same chromosome tend to have higher correlations than randomly selected probes. To exploit the spatial dependency of CGH probes, we devised a recursive algorithm to partition each chromosome into correlated segments using the CNV data. The algorithm is described in Additional file 1, Text S1. 86 segments were obtained from the CNV data and reported in Additional file 6, Table S5. The CNV data of a segment is the mean over the CNV data of all its constituent probes.

### Logistic regression models

We used logistic regressions to model the effects of molecular aberrations on gene or microRNA expressions. Denote *y *the expression of a gene or microRNA and *x *the driver aberrations that explain *y*. The conditional probability is

(1) $$P\left(y|x\right)=\frac{1}{Z\left(x\right)}e^{\sum_{i}\lambda_{i}f_{i}\left(x\right)y},\lambda_{i}\geq 0,\forall i.$$

*f*_*i*_(*x*)'s are scalar feature functions specifying the relations of *x *and *y*. λ_*i*_'s are nonnegative parameters, and *Z*(*x*) is the partition function that normalizes the conditional probabilities. In this work *f*_*i*_(*x*)'s are linear functions of feature values. *f*_*i*_(*x*_*i*_) = *x*_*i *_if aberration *x*_*i *_activates expression *y*, *fi*(*x*_*i*_) = -*x*_*i *_if aberration *x *represses expression *y*.

Given observed data *D *and two nested models *M*_0_,*M*_1 _⊇ *M*_0_, we incurred a standard hypothesis testing procedure to calculate the log-likelihood ratio and p-value of *D*. The pairwise scores measured the goodness of fit of *M*_1 _against the null model *M*_0_. Furthermore, given the models *M*_1 _and *M*_2 _of two candidate driver aberrations we tested the joint model *M*_12 _against *M*_1 _and *M*_2 _respectively. The testing results provide information whether the explanatory power of one model can be covered by another. Detailed procedures of parameter estimation, evaluation of log likelihood ratios and p-values as well as model selection are described in Additional file 1, Text S1.

### Building association modules

An association module consists of a small number of bona fide driver molecular aberrations and a subset of passenger genes or microRNAs. The expression profiles of passengers are explained by fluctuations of drivers. For trans-acting effects with segment CNVs, the associations between drivers and passengers are mediated by regulators on the segments. We require that a valid association module should satisfy the following conditions. First, a driver explains each passenger expression with a significant pairwise score. Second, a driver provides an explanatory power that cannot be replaced by any other drivers. Third, cis-acting effects have a higher priority than associations with non-local aberrations. Fourth, a module has at least 10 passengers. Toward this goal the association modules were constructed by the following procedures.

1. For each passenger mRNA or microRNA expression, evaluate the pairwise association scores with all candidate driver aberrations. Keep the associations that pass the threshold values of these scores.

2. If a passenger gene or microRNA is associated with an external segment CNV, then find the transcription factor mediating the associations between external segment CNVs and passenger expressions.

3. Rule out a transcription factor as a candidate driver for the modules of regulatory effects if the transcription factor is associated with any observed molecular aberration.

4. For each passenger mRNA or microRNA expression, incur model selection to rule out the driver aberrations that can be replaced by other drivers. Briefly, for the models *M*_1 _and *M*_2 _of two candidate driver aberrations, test the joint model *M*_12 _against *M*_1 _and *M*_2 _respectively. If *M*_12 _is significantly better than *M*_1 _but does not outperform *M*_2_, then remove *M*_1_. Apply this filtering procedure for all pairs of driver aberrations.

5. Group passenger genes or microRNAs into modules by their drivers. Report the modules with ≥ 10 passenger members. Passengers of distinct association modules may overlap.

Detailed procedures for building association modules are described in Additional file 1, Text S1.

### Experimental validation

#### Selection of putative targets and control genes of MYB

To validate the causal implications of association outcomes we selected several putative targets of MYB and measured their expression responses with and without the treatment with an MYB siRNA. Putative MYB targets satisfy the following criteria: (1)their mRNA expressions were positively associated with segment 31 CNV, (2)their mRNA expressions were positively associated with MYB expression in NCI-60 data under normal conditions, (3)their mRNA expressions were positively associated with MYB expression in another expression dataset of NCI-60 cell lines exposed under radiation ([53], Additional file 1, Figure S19), (4)their mRNA expressions were positively associated with MYB in a dataset containing 73 normal tissues [54]. 31 genes passed these filtering criteria. Among them we then selected 11 genes based on the constraints of primer design: CPSF6, CTCF, HCLS1, KHDRBS1, NFATC3, ORC1L, PAICS, POLD3, RBMX, SMC1A, and ZNF131.

In addition to predicted MYB targets we also selected several control genes which were constituently expressed across the NCI-60 cell lines, regardless of the MYB expression levels. From the top 10 candidates we selected 6 based on the constraints of primer design: SSR2, GNB2, HSPC152, EIF4A1, RPS15, HINT1. Detailed procedures of selecting putative MYB targets and control genes are described in Additional file 1, Text S1.

#### siRNA treatments and RNA measurements

Human erythroleukemia cells (K562) were purchased from NCI (Frederick, MD) and maintained in RPMI 1640 medium (Invitrogen, CA). The sequences of siRNAs were adopted from previous reports: siRNA against c-myb (c-myb siRNA); sense: 5-UGUUAUUGCCAAGCACUUAAA -3; anti-sense: 5-UAAGUGCUUGGCAAUAACAGAA -3; siRNA against GFP (control siRNA): sense sequence: 5-UGCGCUCCUGGACGUAGCCTT-3; antisense: 5-GGCUACGUCCAGGAGCGCATT-3.

siRNA transfection was performed as described previously with minor modifications. Briefly, cells were seeded at a density of 2 × 10^5 ^cells/well in a 6-well plate with 2 mL culture medium per well. Right after the seeding, the cells were transfected with c-myb siRNA or control siRNA using LipofectAMINE 2000 (Lf 2000, Invitrogen, CA). The siRNA/Lf2000 complex was then incubated with the cells (siRNA concentration = 100 nM) for 48 h and the cells were harvested for further assays.

RNA isolation and cDNA synthesis were demonstrated according to the manufacturers protocols. Briefly, total RNA of the treated K562 cells was isolated and then converted to cDNA by incubating 1 *μ*g RNA with 2.5 *μ*M Oligo(dT)23, 500 *μ*M dNTP and 1 *μ*l of RNase Inhibitor (New England Biolabs, Beverly, MA), 5 mM DDT, 4 *μ*l of 5 First-Strand buffer and 1 *μ*l of SuperScript III Reverse transcriptase (Invitrogen) for 1 h at 50 degrees Celsius in a total volume of 20 *μ*l in a microtube.

Real-time PCR was performed in a Mastercycler ep-gradient-S thermocycler (Eppendorf, Hamburg, Germany) with FastStart TaqMan Probe Master (ROX) and Universal ProbeLibrary (Roche Diagnostics GmbH, Manheim, Germany) according to the manufacturers instructions. The primers and the probe used for detecting each gene are listed in Additional file 8, Table S7. The amplification conditions were 10 min at 95 degrees Celsius, followed by 40 cycles of 95 degrees Celsius for 15 s and 60 degrees Celsius for 1 min. The quantity was determined from the experimental threshold cycle on a standard curve of the data from a series of serial dilution of the mixture of generated cDN A. The mRN A level of the gene of interest was normalized by that of ACTB (*β*-actin) as an endogenous control. Detailed procedures of RNA quantification are described in Additional file 1, Text S1. The measured data of qRT-PCR are reported in Additional file 7, Table S6.

### In-silico validation

#### Evaluation of false discovery rates

For each type of association modules, we randomly permuted aberration and expression data 1000 times and estimated the empirical distribution of the number of significant pairwise associations arising from permuted data. The expected number and the 99 percentile number of false positives were calculated from this empirical distribution. The number of positive calls from the data was the number of significant pairwise associations. Two types of false discovery rates were evaluated accordingly: $\left(1\right)\;\frac{\text{expected \# false positives according to the null model}}{\#\text{positive calls from the date}},\;\left(2\right)\;\frac{\#\text{ false positives in the 99 percentile of the null model}}{\#\text{ positive calls from the date}}$.

#### Enrichment analysis of putative targets on passenger genes

19 transcription factors appear in both drivers/regulators of the association modules and the TRANSFAC database [37]. We extracted their binding motifs from TRANSFAC and 5kb promoter sequences of 27748 human genes from the UCSC Genome Browser [55]. For each transcription factor, the occurrences of its binding motif on all promoters and on the passenger promoters were counted. A standard Fisher's exact test was applied to evaluate the significance of motif enrichment on passenger genes. Motif search and Fisher's exact test were implemented by our own C programs.

Two association modules of mRNA expressions contain microRNA expressions as drivers. We extracted the putative targets of the driver microRNAs from the union of three databases: TargetScan [38], microRNA.org [39], and miRBase [40]. Enrichment analysis was carried out on the passenger genes of these modules.

#### Co-citation analysis on PubMed database

We incurred a batch search on the PubMed database to find all the pairs of drivers/regulators and passengers that were co-cited in the same publications. The spurious results from the automated search were removed by human inspection. Manual curation also identified the pairs conferring regulatory or association relations. To assess the confidence of co-citation outcomes, for each module we replaced passengers with random genes or microRNAs and counted co-cited pairs. The maximum numbers of co-cited pairs over 10 random trials are reported in Table 4.

#### Enrichment analysis of functional categories and pathways

We extracted 4822 functional categories from the Gene Ontology database [41] and 889 pathways from three pathway databases: Reactome [43], BioCarta [44], and the NCI Pathway Interaction Database [42]. For each association module, we applied standard a Fisher's exact test to identify enriched GO categories and pathways for the passenger genes.

### Extraction of tissue-specific patterns

We extracted tissue-specific patterns of association modules with the following procedures. First, we obtained the tissue-specific pattern for each mRNA expression profile. An expression profile was written as a linear combination of "ideal" tissue-specific expression profiles, and the up/down regulation on a tissue type was determined by its mixture coefficient. An ideal tissue-specific expression profile has 1s on samples belonging to the target tissue and 0s on all other samples. Second, for each association module we checked whether its passengers were enriched with tissue-specific genes of each tissue type. Third, we employed gene set enrichment analysis [21] to find tissue-specific patterns of drivers. Finally we reported the intersection of tissue-specific patterns of drivers and passengers. Detailed procedures are elaborated in Additional file 1, Text S1.

## Authors' contributions

CHY conceived and encoded the association algorithms and performed computational analysis on NCI-60 data. SDL and TT performed the siRNA experiments on MYB targets. YFH performed co-citation analysis on inferred modules. All authors read and approved the final manuscript.

## Supplementary Material

Additional file 1 **Text S1 includes procedures of data processing, model selection, experimental and in-silico validations, heatmap visualizations of molecular aberrations and mRNA/microRNA expressions of association modules, and distributions of correlation coefficients between segment CNVs and their constituent genes and microRNAs**. Click here for file

Additional file 2 **Table S1 reports the association modules of mRNA expressions**. Click here for file

Additional file 3 **Table S2 reports the association modules of microRNA expressions**. Click here for file

Additional file 4 **Table S3 reports the co-cited driver/regulator-passenger pairs and the PubMed IDs of the citations in association modules**. Click here for file

Additional file 5 **Table S4 reports the enriched GO categories and pathways for each association module**. Click here for file

Additional file 6 **Table S5 reports the information of partitioned segments**. Click here for file

Additional file 7 **Table S6 reports the expression responses of putative targets and control genes in c-myb siRNA experiments**. Click here for file

Additional file 8 **Table S7 reports the primer sequences for RT-PCR in c-myb siRNA experiments**. Click here for file

Additional file 9 **Table S8 reports the enrichment of driver/regulator binding motifs on passenger promoters with multiple motif occurrences**. Click here for file

Additional file 10 **Table S9 reports the association outcomes between candidate drivers and GO categories/pathways**. Click here for file
