# Supplementary material for: Deciphering causal and statistical relations of molecular aberrations and gene expressions in NCI-60 cell lines
Source: BMC Syst Biol. 2011 Nov 4;5:186. doi: 10.1186/1752-0509-5-186 (PMC3259106; doi:10.1186/1752-0509-5-186)

Deciphering causal and statistical relations of molecular aberrations and gene expressions in NCI-60 cell lines. Supplementary Materials.

Shyh-Dar Li[2], Tatsuaki Tagami[3], Ying-Fu Ho[1], Chen-Hsiang Yeang[1]*

[1]Institute of Statistical Science, Academia Sinica, Taipei, Taiwan.

[2]Ontario Institute for Cancer Research, Toronto, Canada.

[3]Nagoya City University, Nagoya, Japan.

*Corresponding Author.

Table of Content

– Data pre-processing procedures.
  - Data sources.
  - Data normalization.
  - Combination of mRNA expression data.
  - Partitioning a chromosome into segments.
  - Clustering DNA methylation, mRNA and microRNA expression data.

– Construction of association modules.
  - Logistic regression models.
  - Model comparison and selection.
  - Building association modules.

– Validation of association modules.
  - FDR evaluation.
  - Experimental validation.
  - In-silico validation.
  - Extraction of tissue-specific patterns

– Heatmap visualizations of mRNA and microRNA association modules.

– mRNA expressions of MYB, its putative targets and control genes under normal condition and radiation treatment.

– Distribution of correlation coefficients between segment CNVs and their constituten genes and microRNAs.

– Tissue-specific patterns of microRNA association modules.

Data pre-processing

Data sources

Seven datasets of NCI-60 cell lines were downloaded from the website of the Genomics and Bioinformatics Group at NCI (GBC): mutation analysis of 24 cancer genes [1], Comparative Genomic Hybridization (CGH) array data of DNA copy number variations [2], cytosine methylation profiling on promoters [3], cDNA microarray data [4], Affymetrix transcript profile data [5], and Agilent transcript profile data [6] of mRNA expressions, and microRNA expression data [7]. The union of these datasets probed 14856 genes and 303 microRNAs.

Chromosomal locations of protein-coding genes in human were downloaded from the NCBI database, and chromosomal locations of microRNAs were downloaded from miRBase [8]. Information about human transcription factors and their targets were obtained from TRANSFAC [9] and FANTOM4 [10] databases. The binding motif sequences of transcription factors were extracted from TRANSFAC. In addition, we obtained the putative targets of microRNAs from the union of three databases: microRNA.org [11], TargetScan [12], and miRBase [8]. In total, 474 of the 14856 genes are transcription factors appeared in TRANSFAC or FANTOM4, and 265 microRNAs contain at least one putative target.

Functional categories and their member genes were downloaded from the GO database [13]. Pathway information was downloaded from three sources: Reactome [14], BioCarta [15], and NCI-Nature database [16]. In total, 4822 GO categories and 889 pathways were examined in enrichment analysis.

Data normalization

To incorporate the diverse NCI-60 datasets in the integrated models we converted them into the same format with compatible scales. Features with numerical values – mRNA and microRNA expressions, copy number variations, DNA methylations – were treated as discrete random variables with three possible states – up-regulation, down-regulation and no change. Features with categorical values – mutations – were treated as binary random variables. Each entry in a data matrix was represented as a probability vector of the underlying discrete states. For instance, if the expression level of a gene in a cell line is high, then Pr(up-regulation) is close to 1, whereas Pr(down-regulation) and Pr(no change) are close to 0.

Mutation data were directly converted into probability vectors by assigning the entire probability mass on the reported states. Thus an entry with value 0 (no mutation) was converted into vector (Pr(no mutation)=1,Pr(mutation)=0), and an entry with value 1 (mutation) was converted into vector (Pr(no mutation)=0,Pr(mutation)=1). In contrast, other types of data require a normalization procedure since we have to convert them into the same scales and preserve the information of continuous values. We applied *probabilistic quantization* to convert measurement values into the trinary probability vectors. Probabilistic quantization transforms continuous variables into discrete variables, which allows a unified model to capture the dependency of these heterogeneous variables. On the other hand, values of continuous measurements are converted into probabilities of discrete states, hence information loss due to hard quantization is avoided. For each dataset, denote z_{ij} the observed value of gene i on cell line j, and x_{ij} its discrete hidden state. The following procedures convert each z_{ij} into a probability vector $(P(x_{ij} = -1), P(x_{ij} = 0), P(x_{ij} = 1))$.

1. Discard the measurement of a gene if there are ≥ 5 missing data points among the 60 cell lines.

2. If z_{ij} has a missing value, then assign equal probability ($P(x_{ij} = -1) = \frac{1}{3}, P(x_{ij} = 0) = \frac{1}{3}, P(x_{ij} = 1) = \frac{1}{3}$) to each state.

3. Rank-transform z_{ij} into the cumulative distribution function (CDF) value $y_{ij} \in [0, 1]$. For the datasets reporting relative values (cDNA, Agilent and CGH data), rank transformation is applied to the entire matrix. For the datasets reporting absolute values (Affymetrix and microRNA data), each feature is rank-transformed separately. This is because we want to capture the relative variation of a feature across different cell lines instead of comparing the values of distinct features. DNA methylation data are scaled in $[0, 1]$ thus need not to be rank-transformed.

4. Convert y_{ij} into a probability vector $(P(x_{ij} = -1), P(x_{ij} = 0), P(x_{ij} = 1))$ with a specific quantization function. Intuitively, a data point with a low CDF value is more likely to be down-regulated ($P(x_{ij} = -1)$ is high), and a data point with a high CDF value is more likely to be up-regulated ($P(x_{ij} = 1)$ is high). This intuition is translated into the requirements that a quantization function is monotonic and maps $y_{ij} = 0$ into $P(x_{ij} = -1) = 1$ and $y_{ij} = 1$ into $P(x_{ij} = 1) = 1$. We chose polynomial functions f_γ and $\bar{f}_\gamma$ as the quantization curves.

$$\begin{aligned} P(x_{ij} = 1|y_{ij}, \gamma) &= f_\gamma(y_{ij}) \equiv y_{ij}^\gamma. \\ P(x_{ij} = -1|y_{ij}, \gamma) &= \bar{f}_\gamma(y_{ij}) \equiv (1 - y_{ij})^\gamma. \\ P(x_{ij} = 0|y_{ij}, \gamma) &= 1 - P(x_{ij} = 1|y_{ij}, \gamma) - P(x_{ij} = -1|y_{ij}, \gamma). \end{aligned} \tag{1}$$

Parameter γ controls the "soft thresholds" of assigning x_{ij} to be +1 or -1. A higher γ lifts the threshold on y_{ij} (and $1 - y_{ij}$) of calling the hidden state x_{ij} to be 1 (and -1). Thus a higher γ raises $P(x_{ij} = 0)$ and reduces $P(x_{ij} = \pm 1)$.

5. Quantization results are sensitive to γ values. To reduce the bias induced by a specific quantization function we assigned weights (prior) on f_γ functions and integrate the transformed values over a family of quantization functions. In this work we chose an exponential prior $e^{-(\gamma-1)}$ and restrict $\gamma \in [1, \infty)$. The averaged quantization outputs are:

$$\begin{aligned} P(x_{ij} = 1|y_{ij}) &= \int_1^\infty e^{-(\gamma-1)} f_\gamma(y_{ij}) d\gamma &&= \frac{y_{ij}}{1 - \log y_{ij}}. \\ P(x_{ij} = -1|y_{ij}) &= \int_1^\infty e^{-(\gamma-1)} \bar{f}_\gamma(y_{ij}) d\gamma &&= \frac{1 - y_{ij}}{1 - \log(1 - y_{ij})}. \\ P(x_{ij} = 0|y_{ij}) &= 1 - P(x_{ij} = 1|y_{ij}) - P(x_{ij} = -1|y_{ij}). \end{aligned} \tag{2}$$

The exponential prior $e^{-(\gamma-1)}$ was chosen for the following reasons. First, large γ values are penalized because they assign the probability mass to $x_{ij} = 0$ for most y_{ij} values. An exponential prior naturally penalizes large γ values. Second, it ensures the existence of the integrals in equation 2. Third, the requirements that $P(x_{ij} = 1|y_{ij} = 1) = 1$ and $P(x_{ij} = -1|y_{ij} = 0) = 1$ are satisfied. Fourth, the most justified single value of γ is $\hat{\gamma} = \frac{\log 3}{\log 2}$ because it assigns an equal probability ($\frac{1}{3}$) for each state when the input CDF $y_{ij} = 0.5$. The marginal quantization curves are indeed similar to the quantization curves generated by $\hat{\gamma}$.

Combination of mRNA expression data

mRNA expressions of NCI-60 cell lines were measured by three distinct platforms: cDNA arrays [4], Affymetrix U95 gene chips [5], and Agilent two-channel microarrays [6]. To ensure the quality of association outcomes, we selected the genes with consistent expression profiles across platforms. First, we filtered out the genes with inconsistent CDF profiles between cDNA and Affymetrix datasets (correlation coefficient < 0.4). However, a gene was retained if it had only one valid expression profile (either cDNA or Affymetrix). Second, for the genes that passed the prior filter, the mRNA expression value of an entry is the average of the corresponding CDF values from cDNA and Affymetrix datasets. For the genes with only one valid expression profiles, we copied the valid cDNA or Affymetrix data to the corresponding rows of the mRNA expression matrix. Third, we filtered out the genes with inconsistent CDF profiles between the combined cDNA/Affymetrix data and the Agilent data (correlation coefficient < 0.4). 6888 genes were retained after these filtering processes.

Partitioning a chromosome into segments

Spatial dependency of CNVs on NCI-60 cell lines was manifested in previous studies [7], [17]. Adjacent probes tend to have correlated copy number variations as they are likely to be included in the same amplification or deletion events. Therefore, instead of associating gene expressions with each CGH probe separately it is more efficient to partition each chromosome into segments with coherent CNV profiles and consider the associations with segment CNVs. Similar approaches of building linkage disequilibrium (LD) blocks have also been adopted in genome-wide association studies.

We proposed a recursive algorithm to partition a chromosome into segments according to the CGH data of spatially sorted probes. In brief, the CGH data of all probes on the same segment are treated as noisy instantiations of a common hidden variable (a naive Bayes model, [18]). The algorithm iteratively partitions a segment that optimizes the joint likelihood and stops when further partitions do not improve the likelihood score.

1. For each chromosome, sort the CGH probes by their coordinates.
2. Apply probabilistic quantization to convert the CGH data into trinary probability vectors.
3. Denote $S = \{\pi_1, \cdots, \pi_m\}$ a collection of m consecutive probes and X_S their normalized data. An entry $X_S(i, j, k) \in X_S$ denotes the probability of probe i on sample j and state k. The log likelihood function $L(X_S)$ of a naive Bayes model of X_S is computed by the following procedures:
   (a) For each sample j evaluate the fractional counts for each state k: $f_j(k) = \sum_{i=1}^{m} X_S(i, j, k)$.
   (b) Choose the state $\hat{k}_j = \arg\max_k f_j(k)$ that maximizes the fractional count on sample j. Assign the state of the hidden variable on sample j to the consensus state over all probes: $y_S(j) = \hat{k}_j$.
   (c) Calculate the prior probabilities of the hidden variable: $p(k) = \frac{\sum_{j=1}^{n} I(y_S(j)=k)}{n}$, where n is the sample size.
   (d) Calculate the transition probabilities of observing a probe state l conditioned on a hidden state k: $q(l|k) = \frac{N(X_S=l, y_S=k)}{N(y_S=k)}$, where $N(X_S = l, y_S = k) \equiv \sum_{j=1}^{n} I(y_S(j) = k) f_j(l)$ and $N(y_S = k) \equiv \sum_{l'=-1}^{1} \sum_{j=1}^{n} I(y_S(j) = k) f_j(l')$.

(e) The log likelihood of the naive Bayes model is $L(X_S) = \sum_{k=-1}^{1} [N(y_S = k) \log p(k) + \sum_{l=-1}^{1} N(X_S = l, y_S = k) \log q(l|k)]$.

4. Initially set S to be the probes of the entire chromosome.
5. Iteratively incur the function $partition(S)$:
   (a) Evaluate $L(X_S)$.
   (b) Find the binary partition (S_1, S_2) of S that maximizes $L(X_{S_1}) + L(X_{S_2})$.
   (c) Stop and return S if $L(X_S) \geq L(X_{S_1}) + L(X_{S_2}) - 10$.
   (d) Otherwise incur $partition(S_1)$ and $partition(S_2)$.

86 segments were generated from the CGH data. The chromosomal coordinates and associated genes/mircoRNAs for each segment are reported in Supplementary Table 5. The CNV data of a segment are the means of the rank-transformed (CDF) values over its constituent probes.

Clustering DNA methylation, mRNA and microRNA expression data

Correlated driver aberrations tend to fit the same set of passenger genes. It is therefore more efficient to cluster the driver aberrations and build association modules with the proxy data derived from clustered driver aberrations. We proposed a graph-based method to cluster DNA methylation, mRNA and microRNA expression data. In brief, by setting a threshold on correlation coefficients we could build a graph with genes as nodes and edges connecting correlated genes. By gradually lowering the threshold we extracted cliques – maximally and completely connected components – with increasing sizes. Highly connected cliques were then merged to form clusters.

1. Calculate the correlation coefficient matrix of the data.
2. Sort the pairwise correlation coefficients with a decreasing order.
3. Set the threshold to the highest correlation coefficient value.
4. Build a graph G with nodes as genes and edges connecting genes whose correlation coefficients exceed the threshold.
5. Find cliques on G.
6. Repeat the following steps until the threshold value ≤ 0.4.
   (a) Lower the threshold value. Add edges to G according to the new threshold value.
   (b) Find all existing clique pairs that become fully connected on the updated G. Among them merge the clique pair with the largest joint size.
   (c) Find the nodes that are connected to existing cliques with the new threshold value. Assign each node to the largest connecting clique.
   (d) Find newly emerged cliques with the new threshold value by incrementally adding nodes with high connectivity.
   (e) Update clique information.
7. Start with cliques as clusters, repeat the following steps until no clusters are mergeable:

(a) For each pair of clusters, calculate the means of intra-cluster and inter-cluster correlation coefficients. Also calculate the mean of the joint cluster correlation coefficient.

(b) Find the cluster pairs whose joint mean correlation coefficient ≥ 0.4 and the difference between the intra-cluster mean correlation coefficient and inter-cluster mean correlation coefficient ≤ 0.1.

(c) Among the candidate cluster pairs merge the ones with the largest joint size.

(d) Update the cluster information.

121, 1530 and 72 clusters were generated from DNA methylation, mRNA and microRNA expression data respectively.

Construction of association modules

Logistic regression models

We used logistic regressions to model the effects of molecular aberrations on gene expressions. Denote y the expression of a gene or microRNA and $\boldsymbol{x}$ the aberrations that explain y. The conditional probability is

$$P(y|\boldsymbol{x}) = \frac{1}{Z(\boldsymbol{x})} e^{\sum_i \lambda_i f_i(\boldsymbol{x}) y}, \lambda_i \geq 0, \forall i. \tag{3}$$

$f_i(\boldsymbol{x})$'s are scalar feature functions specifying the relations of $\boldsymbol{x}$ and y. λ_i's are nonnegative parameters, and $Z(\boldsymbol{x})$ is the partition function that normalizes the conditional probabilities. In this work $f_i(\boldsymbol{x})$'s are linear functions of feature values. $f_i(x_i) = x_i$ if aberration x_i activates expression y, $f_i(x_i) = -x_i$ if aberration x represses expression y.

The log likelihood of a logistic regression model has the following form:

$$\begin{aligned} L(\boldsymbol{x}, y) &= \textstyle\sum_{k=1}^{n} \{\log(P(\boldsymbol{x}_k)) + \log(\frac{1}{Z(\boldsymbol{x}_k)}) + \sum_i \lambda_i f_i(\boldsymbol{x}_k) y_k\} \\ &= \textstyle\sum_{C_{\boldsymbol{x}}, C_y} \{N(C_{\boldsymbol{x}}) \log(P(C_{\boldsymbol{x}})) + N(C_{\boldsymbol{x}}, C_y)[-\log(Z(C_{\boldsymbol{x}})) + \sum_i \lambda_i f_i(C_{\boldsymbol{x}}) C_y]\}. \end{aligned} \tag{4}$$

where $C_{\boldsymbol{x}}$ and C_y stand for configurations of $\boldsymbol{x}$ and y, and $N(C_{\boldsymbol{x}})$, $N(C_{\boldsymbol{x}}, C_y)$ the fractional counts for configurations $C_{\boldsymbol{x}}$ and $(C_{\boldsymbol{x}}, C_y)$ over all samples. Using probabilistic quantization each entry x_{ij} was converted into $(P(x_{ij} = -1), P(x_{ij} = 0), P(x_{ij} = 1))$, where i and j are gene and sample indices. The fractional count for a state configuration $(C_{\boldsymbol{x}}, C_y)$ is

$$N(C_{\boldsymbol{x}}, C_y) = \textstyle\sum_j P(y_j = C_y) \prod_i P(x_{ij} = C_{\boldsymbol{x}}(i)). \tag{5}$$

The maximum likelihood parameters of λ_i's were numerically estimated using the Newton-Raphson method.

Given observed data D and two nested models $M_0, M_1 \supseteq M_0$, we incurred a standard hypothesis testing procedure to calculate the log-likelihood ratio and χ^2 p-value:

$$\begin{aligned} \mathcal{L}(D; M_0, M_1) &= L(D|M_1) - L(D|M_0). \\ p &= 1 - \chi^2_d(2\mathcal{L}(D; M_0, M_1)). \end{aligned} \tag{6}$$

where χ^2_d is the χ^2 CDF function with d degree of freedom. Here d is the number of additional features in M_1 compared to M_0.

The χ^2 p-values tend to over-estimate the significance of the testing results. Thus we also evaluated the p-values of permutation tests and reported the supremum of of χ^2 and permutation p-values. Permutation p-values were calculated by the following procedures:

1. Quantize the aberration and expression CDF values into binary or trinary states. For trinary variables we chose 0.4 and 0.6 as thresholds.
2. Count the number of samples where the aberration and expression states are consistent with the truth tables from M_1. Denote this number as n_C.
3. Find the additional aberrations $X_v \subseteq M_1 \backslash M_0$.
4. Repeat the following steps 10000 times:
   (a) Randomly permute the data in X_v and fix the remaining variables.
   (b) Count the number n_P of samples where the permuted aberration and expression states are consistent with M_1.
5. The p-value is the fraction of n_P's exceeding n_C.

Model comparison and selection

For each (driver,passenger) pair, we can evaluate the log likelihood ratio and p-value of the data. The null model M_0 assumes the driver and the passenger are independent, whereas the alternative model M_1 adds a (positive or negative) link between them in a logistic regression model. Pairwise scores measure the goodness of fit to the observed data but do not provide explicit information about dependencies between multiple candidate aberrations. These candidates may all exhibit high pairwise scores, yet the effects from some of them may be mediated by other candidates. It is of interest to distinguish direct from indirect effects. Previous studies addressed this issue by a variety of statistical tools such as conditional independence [19] and information processing inequality [20]. In this work we provide a solution under the hypothesis testing framework.

Consider two candidate aberrations x_1 and x_2 that can both explain gene expression y. We built two logistic regression models M_1 and M_2 with y as the output variable and x_1 and x_2 as input variables respectively. A joint model M_{12} contains both x_1 and x_2 as input variables, and a null model M_0 assumes y independent of x_1 and x_2. We are interested in the candidate aberrations with strong pairwise scores. Thus both M_1 and M_2 can significantly fit the data better than M_0. We then tested M_{12} against M_1 and M_2. The following operations were executed depending on the testing outcomes:

1. M_{12} is significantly better than M_1 but not significantly better than M_2. M_1 does not provide an additional explanatory power to M_2, but M_2 provides an additional explanatory power to M_1. Thus the effect of M_2 dominates the effect of M_1. Keep M_2 and remove M_1.
2. M_{12} is significantly better than M_2 but not significantly better than M_1. Keep M_1 and remove M_2 for the same reason.

3. M_{12} is significantly better than both M_1 and M_2. Each model provides an additional explanatory power to another. Keep both M_1 and M_2.
4. M_{12} is significantly better than neither M_1 nor M_2. Neither model provides an additional explanatory power to another. The two models are redundant and one of them suffices to fit the data. Keep the one with a higher pairwise score.

Building association modules

An association module consists of subsets of driver aberrations (segment CNVs, mutations, DNA methylations, microRNA and transcription factor expressions), passenger mRNAs or microRNAs, and regulators (transcription factors) that mediate the effects from drivers to passenger expressions. We require that a valid association module should satisfy the following conditions. First, a driver yields a significant pairwise score to explain each passenger expression. Second, a driver provides an explanatory power that cannot be replaced by any other drivers. Third, cis-acting effects have a higher priority than associations with non-local aberrations. Fourth, the number of passengers in a module exceeds a threshold value. Toward this goal the following procedures were adopted to construct association modules.

1. For each passenger mRNA or microRNA expression, evaluate the pairwise association scores with the following types of molecular aberrations: one local segment CNV, 85 external segment CNVs, mutations of 24 genes, DNA methylations of 121 gene clusters, microRNA expressions of 72 cliques, mRNA expressions of 235 transcription factors. Apply the following thresholds of pairwise scores: local segment CNVs – correlation coefficient 0.3, log likelihood ratio 7.0, p-value 0.05; external segment CNVs and transcription factor expressions – correlation coefficient 0.3, log likelihood ratio 7.0, p-value 0.02; mutations – correlation coefficient 0.3, log likelihood ratio 7.0, p-value 0.005; DNA methylation – correlation coefficient -0.3, log likelihood ratio 7.0, p-value 0.005; microRNA expression – correlation coefficient -0.3, log likelihood ratio 7.0, p-value 0.005. If the passenger is a microRNA, then do not perform associations with other microRNA expressions. Keep the associations passing the thresholds.
2. Rule out a transcription factor as a candidate regulator for the modules of regulatory effects if the transcription factor is associated with any observed molecular aberration.
3. If a passenger gene or microRNA is associated with an external segment CNV, then find the intermediate regulators satisfying the following conditions: (1)the transcription factor is located on the external segment, (2)the transcription factor has valid mRNA expression data, (3)the transcription factor mRNA is associated with its local segment CNV (i.e., the external segment CNV of the target passenger, correlation coefficient ≥ 0.3, log likelihood ratio ≥ 7.0, p-value ≤ 0.02), (4)the transcription factor mRNA is associated with the passenger expression (with the same thresholds). Discard an association with external segment CNV if intermediate regulators are not found.
4. For each passenger mRNA or microRNA expression, incur the aforementioned model selection procedures to filter out the driver aberrations that can be replaced by other drivers. For each pair of associations M_1 and M_2, build a joint model M_{12} and test M_{12} against M_1 and M_2. Remove M_1 if M_{12} is significantly better than M_1

and not significantly better than M_2. The thresholds of log likelihood ratios and p-values of the tests are 1.5 and 0.1 respectively.

5. However, an association with a local segment CNV is retained regardless of the testing outcomes with other associations.
6. Group passenger genes or microRNAs into modules by their drivers. Report the modules with ≥ 10 passenger members.

Validation of association modules

FDR evaluation

False discovery rates (FDRs) quantify the expected fraction of false positives among the positive calls from a multiple-hypothesis testing problem. To simplify the testing procedures, we considered a positive call as a significant pairwise association between a driver and a passenger. The thresholds for significance on correlation coefficients, log-likelihood ratios and p-values were identical to the values applied in building association modules. False positives were positive calls generated from a null model. Lacking specific information about the distribution of noise, we created a simple null model by randomly permuting aberration and expression data 1000 times. Such permutation tests are widely used in evaluating the significance of detected signals. The empirical distribution of the number of significant pairwise associations arising from permuted data provides a reasonable measure for false positive numbers. We characterized false positives with two numbers: (1)the expected number of significant calls according to the distribution from permutation tests, (2)the number of the 99 percentile according to the distribution from permutation tests. The former is commonly adopted in FDR evaluation such as [22], while the latter provides a more conservative estimate and is proposed in [23]. Two types of false discovery rates were evaluated accordingly: (1) $\frac{\text{expected \# false positives according to the null model}}{\text{\# positive calls from the data}}$, (2) $\frac{\text{\# false positives in the 99 percentile of the null model}}{\text{\# positive calls from the data}}$.

Our method considerably differs from the method proposed in [21], which explicitly controlled the FDR value and incurred a Bonferroni-like test on sorted p-values. This method is inadquate for our application, since we use FDR to justify the overall accuracy of inferred models rather than controlling a pre-selected FDR value to curb model selection. In contrast, our method is more akin to the method proposed in [22], which evaluates the FDR on a fixed list of significant calls. The difference between our approach and [22] lies on the estimation of false positive numbers. The method in [22] relies on the uniform distribution of null p-values to estimate false positive numbers. This property may not hold for datasets with many highly dependent features. In contrast, our method applies a simple permutation test without assumptions on null model distributions. It can thus handle the data dependency problems better than [22].

Experimental validation

Selection of putative targets and control genes of MYB To validate the causal implications of association outcomes we selected several putative targets of MYB and measured their expression responses with and without the

treatment with an MYB siRNA. Putative MYB targets satisfied the following criteria: (1)their mRNA expressions were positively associated with segment 31 CNV (correlation coefficient ≥ 0.2, log likelihood ratio ≥ 7.0, p-value ≤ 0.02), (2)their mRNA expressions were positively associated with MYB expression (correlation coefficient ≥ 0.2, log likelihood ratio ≥ 7.0, p-value ≤ 0.1), (3)their mRNA expressions were positively associated with MYB expression in another NCI-60 dataset under the radiation treatment [24] (correlation coefficient ≥ 0.2, log likelihood ratio ≥ 4.0, p-value ≤ 0.05), (4)their mRNA expressions were positively associated with MYB in a dataset containing 73 normal tissues [25] (correlation coefficient ≥ 0.4). 31 genes passed these filtering criteria. Among them we then selected 11 genes based on the constraints of primer design.

In addition to predicted MYB targets we also selected several control genes whose expressions were not anticipated to be affected by MYB knock-down. The control genes were constituently expressed across the NCI-60 cell lines. Therefore, we sorted genes according to their top 25% expression values over the 60 cell lines from two expression datasets ([4] and [5]). From the top 10 genes we selected 6 based on the constraints of primer design.

Cell culture Human erythroleukemia cells (K562) were purchased from NCI (Frederick, MD). The cells were maintained in RPMI 1640 medium (Invitrogen, CA), supplemented with 10% heat-inactivated fetal bovine serum (Invitrogen, CA), 10 mM L-glutamine, 100 U/ml penicillin and 100 μg/ml streptomycin. Cells were incubated at 37 degrees Celcius in a humidified atmosphere of 5% CO_2/95% air incubator.

siRNA siRNA was chemically synthesized and purified by Dharmacon (Lafayette, CO). The sequences of siRNAs were adopted from previous reports: siRNA against c-myb (c-myb siRNA); sense: 5-UGUUAUUGCCAAGCACUUAAA-3; antisense: 5-UAAGUGCUUGGCAAUAACAGAA-3; siRNA against GFP (control siRNA) : sense sequence: 5-UGCGCUCCUGGACGUAGCCTT-3; antisense: 5-GGCUACGUCCAGGAGCGCATT-3.

siRNA transfection siRNA transfection was performed as described previously with minor modifications . Briefly, cells were seeded at a density of 2×10^5 cells/well in a 6-well plate with 2 mL culture medium per well. Right after the seeding, the cells were transfected with c-myb siRNA or control siRNA using LipofectAMINE 2000 (Lf 2000, Invitrogen, CA). siRNA was premixed with Lf 2000 at the ratio of 1 to 5 (siRNA (μg)/Lf2000 (μl)) in Opti-MEM I (Invitrogen) in a microtube, and the mixture was incubated at room temperature for 20 min to form complex. The siRNA/Lf2000 complex was then incubated with the cells (siRNA concentration = 100 nM) for 48 h and the cells were harvested for further assays as described below.

RNA isolation, cDNA synthesis and real-time PCR (quantitative RT-PCR) RNA isolation and cDNA synthesis were demonstrated according to the manufacturers protocols. Briefly, total RNA of the treated K562 cells was isolated using the RNeasy Mini Kit with the RNase-Free DNase Set (Qiagen, Hilden, Germany). Then, theRNA was converted to cDNA by incubating 1 μg RNA with 2.5 μM Oligo(dT)23, 500 μM dNTP and 1 μl of RNase Inhibitor (New England Biolabs, Beverly, MA), 5 mM DDT, 4 μl of 5 First-Strand buffer and 1 μl of SuperScript

III Reverse transcriptase (Invitrogen) for 1 h at 50 degrees Celcius in a total volume of 20 μl in a microtube. The samples were incubated for 10 min at 70 degrees Celcius to inactivate the reverse transcriptase.

Real-time PCR was performed in a Mastercycler ep-gradient-S thermocycler (Eppendorf, Hamburg, Germany) with FastStart TaqMan Probe Master (ROX) and Universal ProbeLibrary (Roche Diagnostics GmbH, Manheim, Germany) according to the manufacturers instructions. Briefly, the PCR mixture was applied in a 96-well plate in a total volume of 20 μl/well including 250 nM probe, 900 nM forward and reverse primers, 2 μl of the generated cDNA and 10 μl of FastStart TaqMan Probe Master (ROX). The set of primers and a probe for real-time RT-PCR were designed using the ProbeFinder software (Roche Diagnostics GmbH). The primers and the probe used for detecting each gene are listed in Supplementary Table 7. The amplification conditions were 10 min at 95 degrees Celcius, followed by 40 cycles of 95 degrees Celcius for 15 s and 60 degrees Celcius for 1 min. The quantity was determined from the experimental threshold cycle on a standard curve of the data from a series of serial dilution of the mixture of generated cDNA. The mRNA level of the gene of interest was normalized by that of ACTB (β-actin) as an endogenous control.

Statistical analysis All values are expressed as the mean ± S.D. Statistical analysis was performed with a two-tailed unpaired t-test using GraphPad Prism software (GraphPad Software, CA). The level of significance was set at p¡0.05.

In-silico validation

Enrichment analysis of putative targets on passenger genes 19 transcription factors appear in both drivers/regulators of the association modules and the TRANSFAC database. We extracted their binding motifs from TRANSFAC and 5kb promoter sequences of 27748 human genes from the UCSC Genome Browser. For each transcription factor, the occurrences of the binding motif on all promoters and on the passenger promoters were counted. A standard Fisher's exact test was applied to evaluate the significance of motif enrichment on passenger genes.

Two association modules of mRNA expressions contain microRNA expressions as drivers. We extracted the putative targets of the driver microRNAs from the union of three databases: TargetScan [12], microRNA.org [11], and the miRBase [8]. Enrichment analysis was carried out on the passenger genes of these modules.

Co-citation analysis We incurred a batch search on the PubMed database to find all the pairs of drivers/regulators and passengers that were co-cited in the same publications. The spurious results from the automated search were removed by human inspection. Manual curation also identified the pairs conferring regulatory or association relations. To assess the confidence of co-citation outcomes, for each module we replaced passengers with random genes or microRNAs and counted co-cited pairs. The maximum numbers of co-cited pairs over 10 random trials are reported.

Functional category and pathway enrichment analysis We extracted 4822 functional categories from the Gene Ontology database [13] and 889 pathways from three pathway databases: Reactome [14], BioCarta [15], and

the NCI-Nature database [16]. For each association module, we applied standard Fisher's exact tests to identify enriched GO categories and pathways for the passenger genes.

Extraction of tissue-specific patterns

We extracted tissue-specific patterns of association modules with the following procedures. First, we obtained the tissue-specific pattern for each mRNA expression profile. We wrote the expression profile of a gene as a linear combination of "ideal" tissue-specific expression profiles:

$$x(t) = \sum_{i=1}^{C} \lambda_i m_i(t) + \delta. \tag{7}$$

where $x(t)$ is the expression profile of a gene and t is the sample index. $x(t)$ is shifted to be within $[-0.5, 0.5]$. For each tissue type i, define an ideal expression profile $m_i(t)$ such that $m_i(t) = 1$ on samples of tissue type i and 0 otherwise. δ is an error term. The mixture coefficients λ_i minimizing the square error $\sum_t (x(t) - \sum_{i=1}^{C} \lambda_i m_i(t))^2$ is

$$\hat{\Lambda} = (MM^T)^{-1}(Mx(t)). \tag{8}$$

where $\hat{\Lambda}$ is the vector of mixture coefficients and M the matrix constituting $m_i(t)$ in each row. A high absolute value of $\hat{\lambda}_i$ implies coherent up/down regulation on tissue type i. To evaluate the significance of $\hat{\lambda}_i$, we randomly permuted the tissue labels of samples 1000 times and counted the fraction of random permutations with higher $|\hat{\lambda}_i|$ than empirical values. We claimed $x(t)$ up-regulated in tissue type i if $\hat{\lambda}_i > 0$ and has p-value ≤ 0.05, and down-regulated in tissue type i if $\hat{\lambda}_i < 0$ and has p-value ≤ 0.05.

Second, for each association module we checked whether its passengers were enriched with genes up/down regulated in specific tissues. Consider a set S_1 of passenger genes for an association module, and a set S_2 of genes up-regulated in tissue type i. We evaluated the hyper-geometric p-value of the enrichment of S_2 in S_1, and claimed that the passenger genes were up-regulated in tissue type i if the hyper-geometric p-value $\leq 10^{-10}$.

Third, for each association module we employed gene set enrichment analysis (GSEA, [26]) to extract tissue-specific patterns of its drivers. Consider a collection of driver aberrations (segment CNVs, DNA methylations, microRNA expressions, TF expressions) of a module and a tissue type i, we want to check the aberrations on tissue type i are significantly up or down regulated among the aberrations of the same drivers on all samples. In GSEA notations, the background list is the driver aberrations on all samples and the gene set is the same driver aberrations on tissue type i. We claimed the driver aberrations significantly up/down regulated in tissue type i if the permutation p-value ≤ 0.05. For mutations, we claimed that the driver aberration states were coherent in tissue type i if the fraction of mutated samples in tissue type i either exceeded 0.7 or was below 0.3.

Fourth, we superimposed the tissue-specific patterns of drivers and passengers for each association module. A module constitutes a tissue-specific pattern in tissue type i if the driver aberrations are coherent in tissue type i and passenger gene expressions are up or down regulated in tissue type i.

References

1. Ikediobi O.N., Davies H., Bignell G., Edkins S., Stevens C., O'Meara S., Santarius T., Avis T., Barthorpe S., Brackenbury L., Buck G., Butler A., Clements J., Cole J., Dicks E., Forbes S., Gray K., Halliday K., Harrison R., Hills K., Hinton J., Hunter C., Jenkinson A., Jones D., Kosmidou V., Lugg R., Menzies A., Mironenko T., Parker A., Perry J., Raine K., Richardson D., Shepherd R., Small A., Smith R., Solomon H., Stephens P., Teague J., Tofts C., Varian J., Webb T., West S., Widaa S., Yates A., Reinhold W., Weinstein J.N., Stratton M.R., Futreal P.A., Wooster R.: **Mutation analysis of 24 known cancer genes in the NCI-60 cell line set.** *Molecular Cancer Therapy* 2006, 5(11):2606-2612.

2. Bussey K.J., Chin K., Lababidi S., Reimers M., Reinhold W.C., Kuo W.L., Gwadry F., Ajay, Kouros-Mehr H., Fridlyand J., Jain A., Collins C., Nishizuka S., Tonon G., Roschke A., Gehlhaus K., Kirsch I., Scudiero D.A., Gray J.W., Weinstein J.N.: **Integrating data on DNA copy number with gene expression levels and drug sensitivities in the NCI-60 cell line panel.** *Molecular Cancer Therapy* 2006, 5(4):853-867.

3. Ehrich M., Turner J., Gibbs P., Lipton L., Giovanneti M., Cantor C., van den Boom D.: **Cytosine methylation profiling of cancer cell lines.** *Proceedings of the National Academy of Science USA* 2008, 105(12):4844-4849.

4. Ross D.T., Scherf U., Eisen M.B., Perou C.M., Rees C., Spellman P., Iyer V., Jeffrey S.S., Van de Rijn M., Waltham M., Pergamenschikov A., Lee J.C., Lashkari D., Shalon D., Myers T.G., Weinstein J.N., Botstein D., Brown P.O.: **Systematic variation in gene expression patterns in human cancer cell lines.** *Nature Genetics* 2000, 24:227-235.

5. Shankarvaram U.T., Reinhold W.C., Nishizuka S., Major S., Morita D., Chary K.K., Reimers M.A., Scherf U., Kahn A., Dolginow D., Cossman J., Kaldjian E.P., Scudiero D.A., Petricoin E., Liotta L., Lee J.K.,: **Transcript and protein expression profiles of the NCI-60 cancer cell panel: an integromic microarray study.** *Molecular Cancer Therapy* 2007, 6(3):820-832.

6. Liu H., D'Andrade P., Fulmer-Smentek S., Lorenzi P., Kohn K.W., Weinstein J.N., Pommier Y., Reinhold W.C.: **mRNA and microRNA expression profiles of the NCI-60 integrated with drug activities.** *Molecular Cancer Therapeutics* 2010, 9(5):1080-1091.

7. Blower P.E., Verducci J.S., Lin S., Zhou J., Chung J.H., Dai Z., Liu C.G., Reinhold W., Lorenzi P.L., Kaldjian E.P., Croce C.M., Weinstein J.N., Sadee W.: **MicroRNA expression profiles for the NCI-60 cancer cell panel.** *Molecular Cancer Therapy* 2007, 6(5):1483-1491.

8. Kozomara A., Griffiths-Jones S.: **miRBase: integrating microRNA annotation and deep-sequencing data.** *Nucleic Acids Research* 2011, 39:D152-D157.

9. Matys V., Fricke E., Geffers R., Gossling E., Haubrock M., Hehl R., Hornischer K., Karas D., Kel AE., Kel-Margoulis OV., Kloos DU., Land S., Lewicki-Potapov B., Michael H., Mnch R., Reuter I., Rotert S., Saxel H., Scheer M., Thiele S., Wingender E. **TRANSFAC: transcriptional regulation, from patterns to profiles.** *Nucleic Acids Research* 2003, 31(1):374-378.

10. Kawaji H., Severin J., Lizio M., Waterhouse A., Katayama S., Irvine K.M., Hume D.A., Forrest A.R., Suzuki H., Carninci P., Hayashizaki Y., Daub C.O.: **The FANTOM web resource: from mammalian transcriptional landscape to its dynamic regulation.** *Genome Biology* 2009, 10(4):R40.

11. Betel D., Wilson M., Gabow A., Marks D., Sander C.: **The microRNA.org resource: targets and expression.** *Nucleic Acids Research* 2008, 36:D149-D153.

12. Lewis B.P., Burge C.B., Bartel D.P.: **Conserved seed pairing, often flanked by adenosines, indicates that thousands of human genes are microRNA targets.** *Cell* 2005, 120(1):15-20.

13. The Gene Ontology Database. http://www.geneontology.org/.
14. Joshi-Tope G., Gillespie M., Vastrik I., D'Eustachio P., Schmidt E., de Bono B., Jassal B., Gopinath G.R., Wu G.R., Matthews L., Lewis S., Birney E., Stein L.: **Reactome: a knowledgebase of biological pathways.** *Nucleic Acids Research* 2005, 33:D428-432.
15. BioCarta Database: http://www.biocarta.com/.
16. Schaefer C.F., Anthony K., Krupa S., Buchoff J., Day M., Hannay T., Buetow K.H." **PID: the Pathway Interaction Database.** *Nucleic Acids Research* 2009, 37:D674-679.
17. Yeang C.H.: **An integrated analysis of molecular aberrations in NCI-60 cell lines** *BMC Bioinformatics* 2010, 11:495.
18. Firedman N., Geiger D. and Goldszmidt M.:**Bayesian network classifiers.** *Machine Learning* 1997, 29:131-163.
19. Schadt E.E., Lamb J., Yang X., Zhu J., Edwards S., Guhathakurta D., Sieberts S.K., Monks S., Reitman M., Zhang C., Lum P.Y., Leonardson A., Thieringer R., Metzger J.M., Yang L., Castle J., Zhu H., Kash S.F., Drake T.A., Sachs A., Lusis A.J.: **An integrative genomics approach to infer causal associations between gene expression and disease.** *Nature Genetics* 2005, 37(7):710-717.
20. Basso L., Margolin A.A., Stolovitzky G., Klein U., Dalla-Favera R., and Califano A.: **Reverse engineering of regulatory networks in human B cells.** *Nature Genetics* 2005, 37(4):382-390.
21. Benjamini Y. and Hochberg Y.: **Controlling the false discovery rate: a practical and powerful approach to multiple testing.** *Journal of the Royal Statistical Society B* 1995, 57(1):289-300.
22. Storey J. and Tibshirani R.: **Statistical significance for genomewide studies.** *Proceedings of the National Academy of Science USA* 2003, 100(16):9440-9445.
23. Korn E.L., Troendle J.F., McShane L.M. and Simon R.: **Controlling the number of false discoveries: application to high-dimensional genomic data.** *Journal of Statistical Planning and Inference* 2004, 124:379-398.
24. Amundson S.A., Do K.T., Vinikoor L.C., Lee R.A., Koch-Paiz C.A., Ahn J., Reimers M., Chen Y., Scudiero D.A., Weinstein J.N., Trent J.M., Bittner M.L., Meltzer P.S., Fornace A.J. Jr.: **Integrating global gene expression and radiation survival parameters across the 60 cell lines of the National Cancer Institute anticancer drug screen.** *Cancer Research* 2008, 68(2):415-424.
25. Su A.I., Wiltshire T., Batalov S., Lapp H., Ching K.A., Block D., Zhang J., Soden R., Hayakawa M., Kreiman G., Cooke M.P., Walker J.R., Hogenesch J.B.: **A gene atlas of the mouse and human protein-encoding transcriptomes.** *Proc. Natl. Acad. Sci. USA* 2004, 101(16):6062-6067.
26. Subramanian A., Tamayo P., Mootha V.K. et al.: **Gene set enrichment analysis: A knowledge-based approach for interpreting genome-wide expression profiles.** Proc. Natl. Acad. Sci. 2005, 102(43):15545-15550.

Figures

Fig. 1. Driver intra-segment CNVs and passenger mRNA expressions across NCI-60 cell lines. Modules 1-10. BR: breast cancer, CNS: central nervous system cancer, CO: colorectal cancer, LE: leukemia, ME: melanoma, LC: lung cancer, OV: ovarian cancer, PR: prostate cancer, RE: renal cancer.

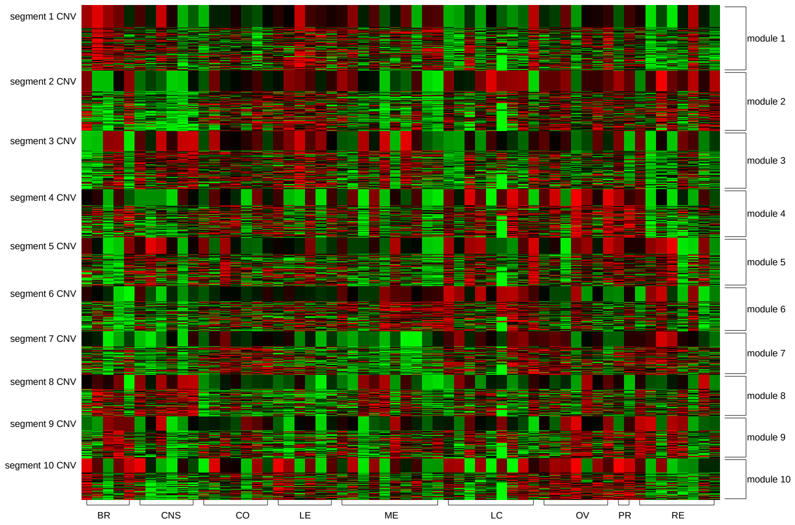

Fig. 2. Driver intra-segment CNVs and passenger mRNA expressions across NCI-60 cell lines. Modules 11-20.

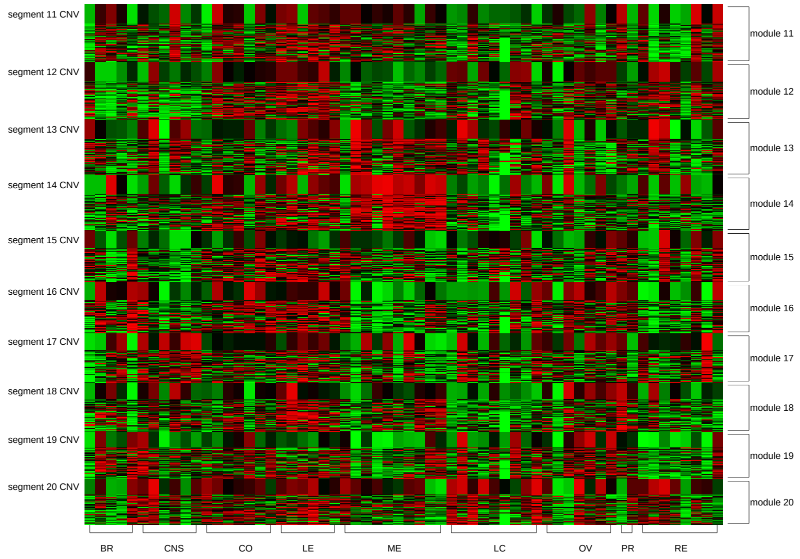

Fig. 3. Driver intra-segment CNVs and passenger mRNA expressions across NCI-60 cell lines. Modules 21-30.

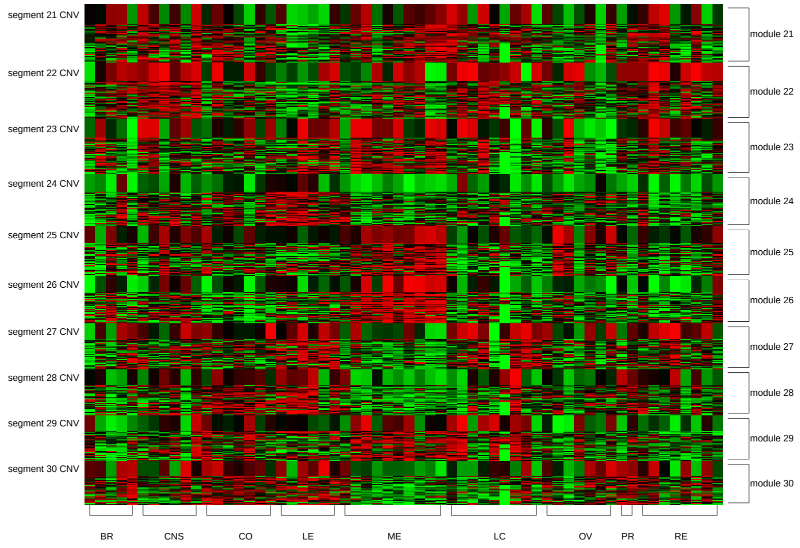

Fig. 4. Driver intra-segment CNVs and passenger mRNA expressions across NCI-60 cell lines. Modules 31-40.

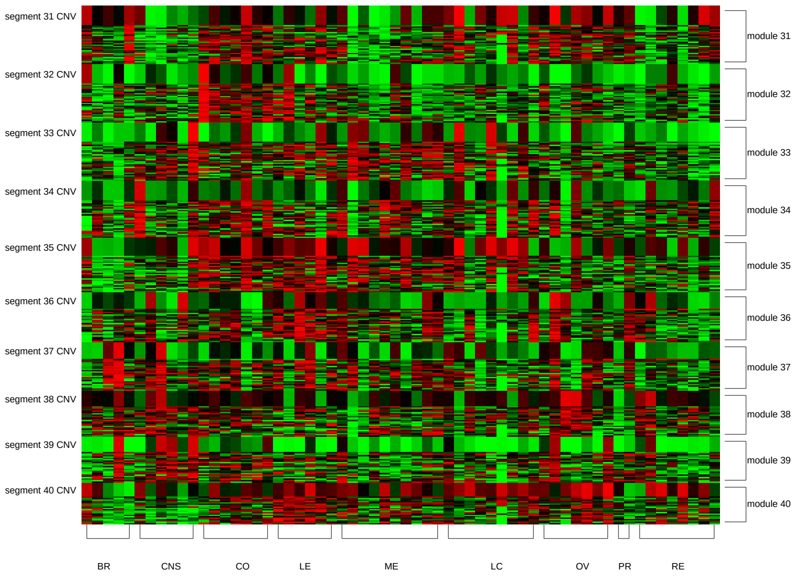

Fig. 5. Driver intra-segment CNVs and passenger mRNA expressions across NCI-60 cell lines. Modules 41-43.

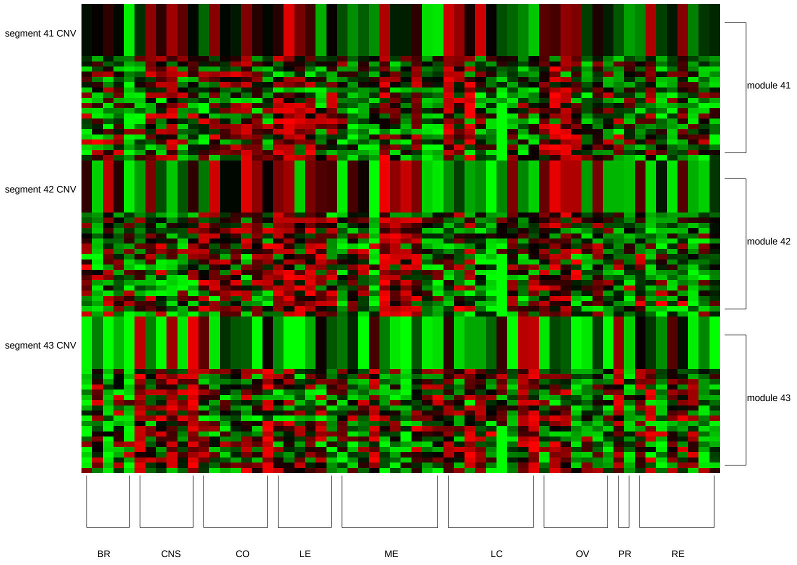

Fig. 6. Driver inter-segment CNVs, regulator and passenger mRNA expressions across NCI-60 cell lines. Modules 44-49.

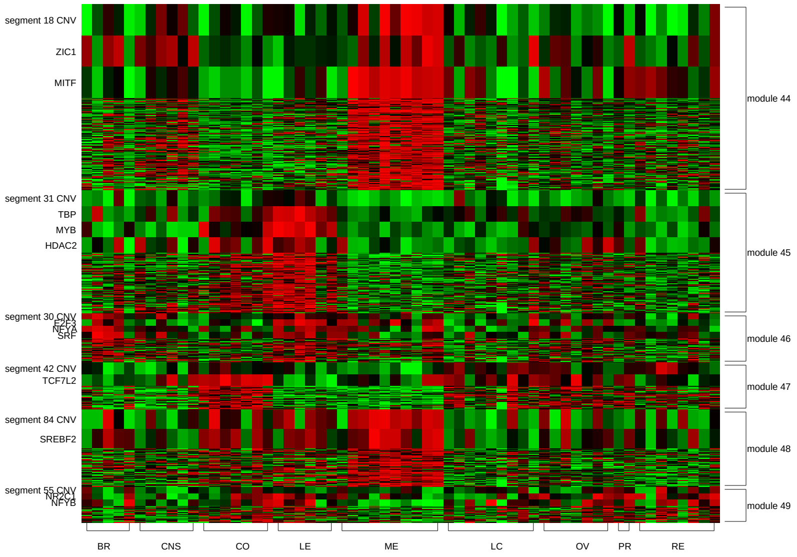

Fig. 7. Driver inter-segment CNVs, regulator and passenger mRNA expressions across NCI-60 cell lines. Modules 50-55.

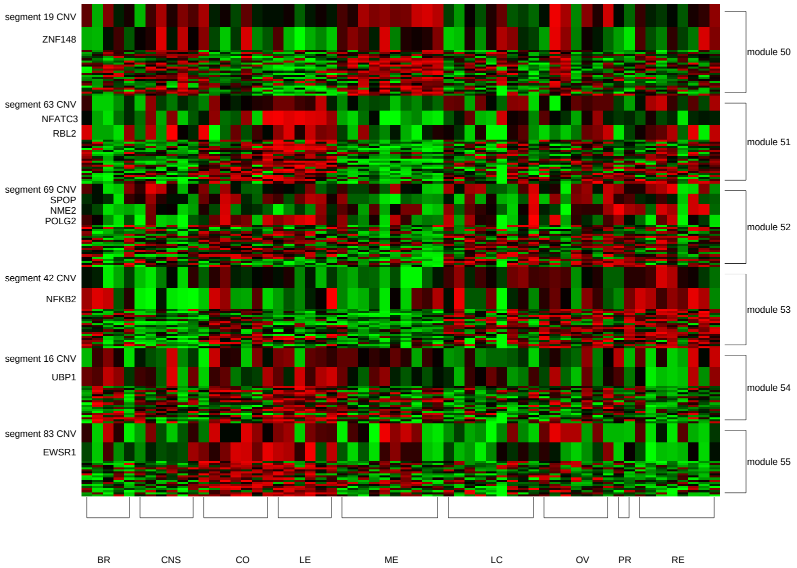

Fig. 8. Driver inter-segment CNVs, regulator and passenger mRNA expressions across NCI-60 cell lines. Modules 56-61.

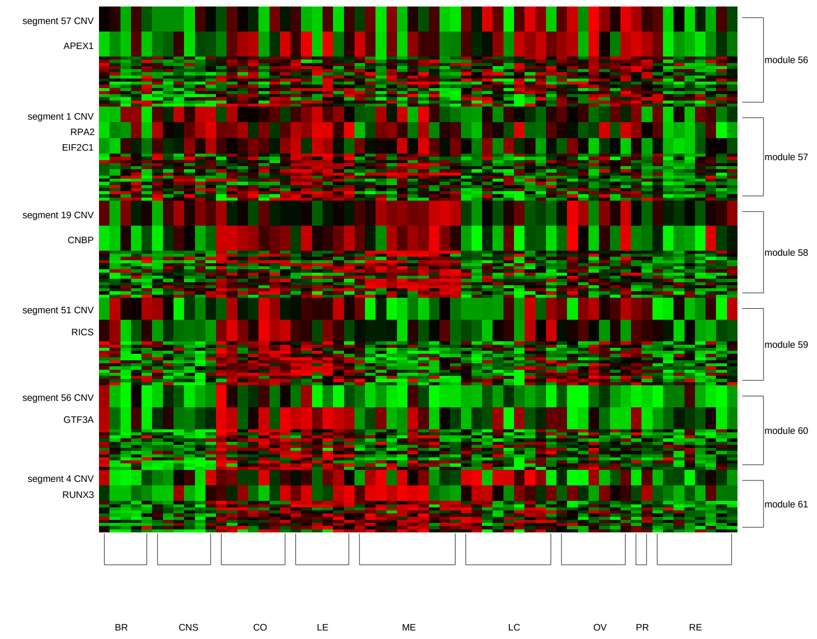

Fig. 9. Driver mutations and passenger mRNA expressions across NCI-60 cell lines. Modules 62-68.

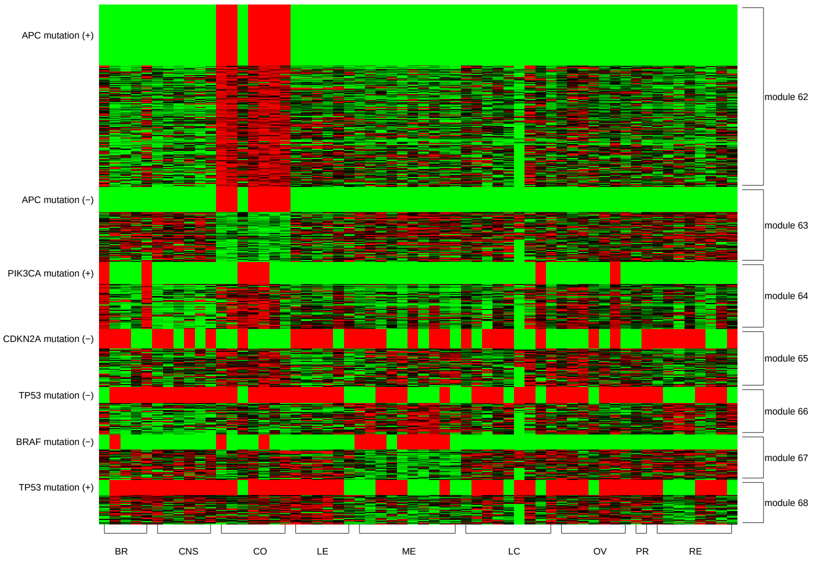

Fig. 10. Driver mutations and passenger mRNA expressions across NCI-60 cell lines. Modules 69-75.

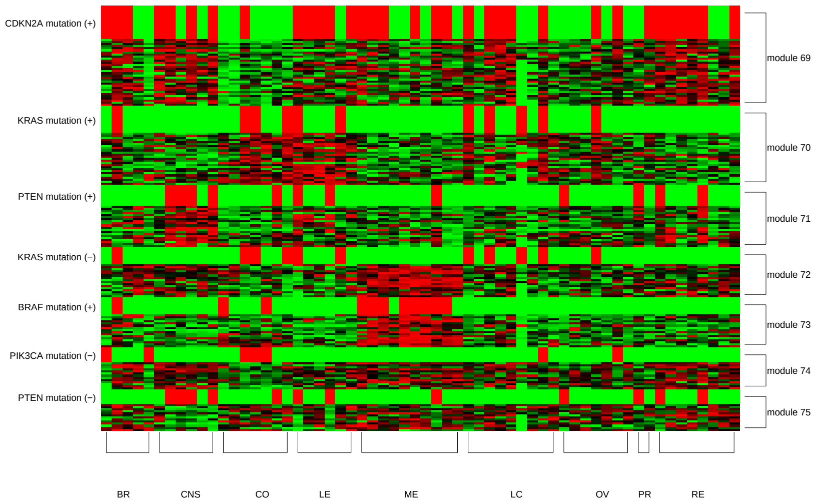

Fig. 11. Driver DNA methylations and passenger mRNA expressions across NCI-60 cell lines. Modules 76-80.

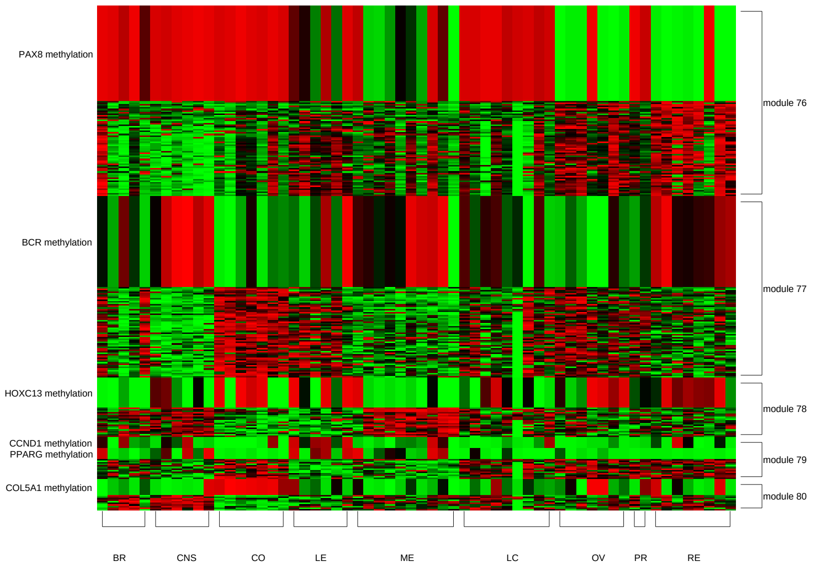

Fig. 12. Driver microRNA expressions and passenger mRNA expressions across NCI-60 cell lines. Modules 81-82.

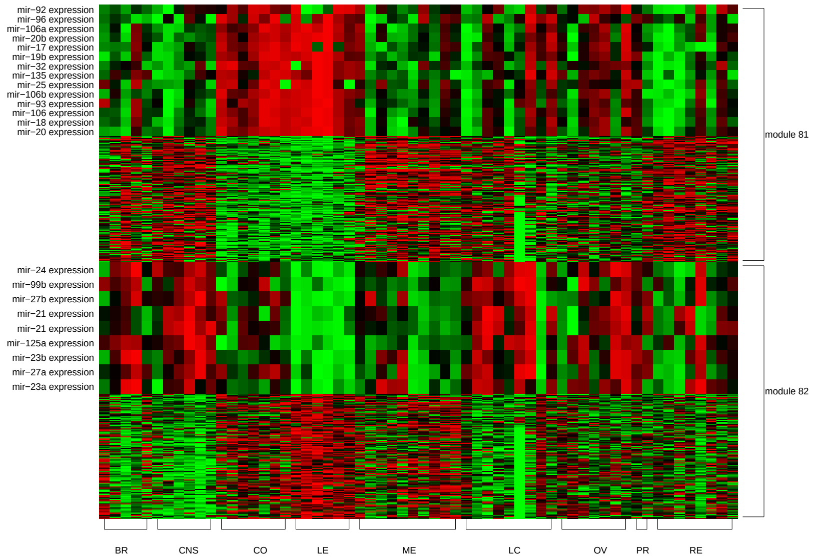

Fig. 13. Driver transcription factor expressions and passenger mRNA expressions across NCI-60 cell lines. Modules 83-84.

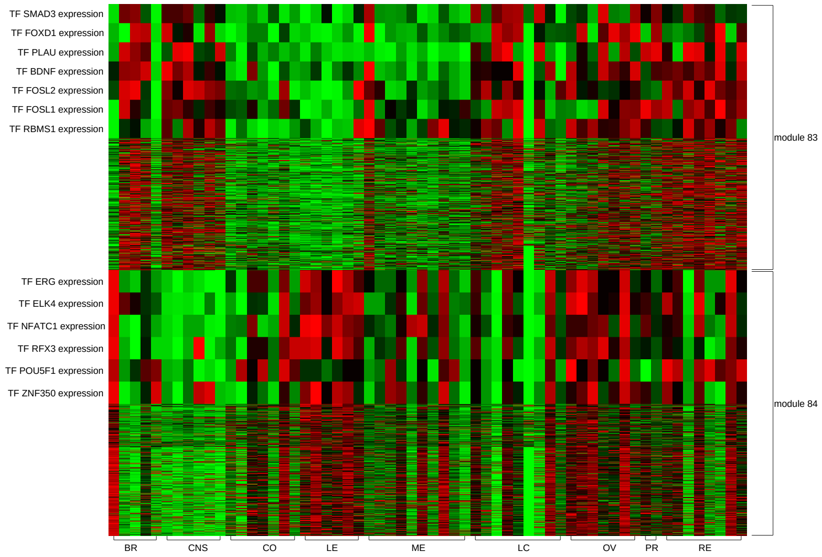

Fig. 14. Driver inter-segment CNVs, regulator mRNA and passenger microRNA expressions across NCI-60 cell lines. Modules 1-9.

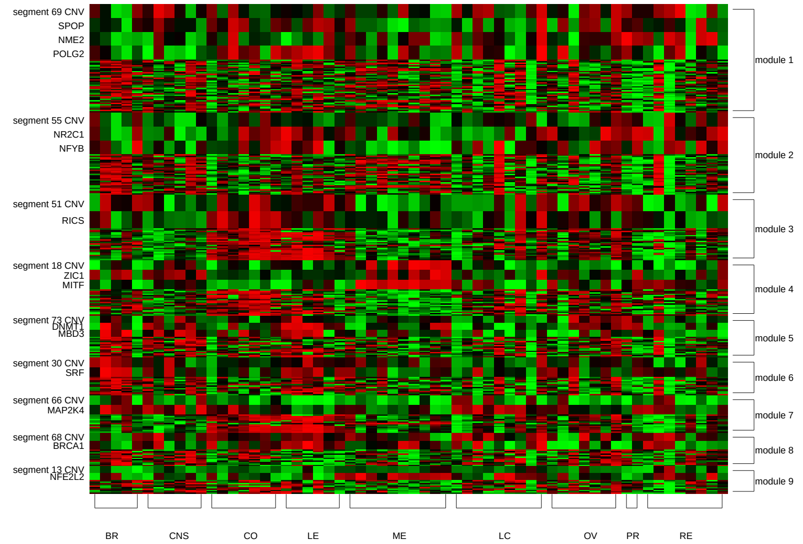

Fig. 15. Driver mutations and passenger microRNA expressions across NCI-60 cell lines. Modules 10-13.

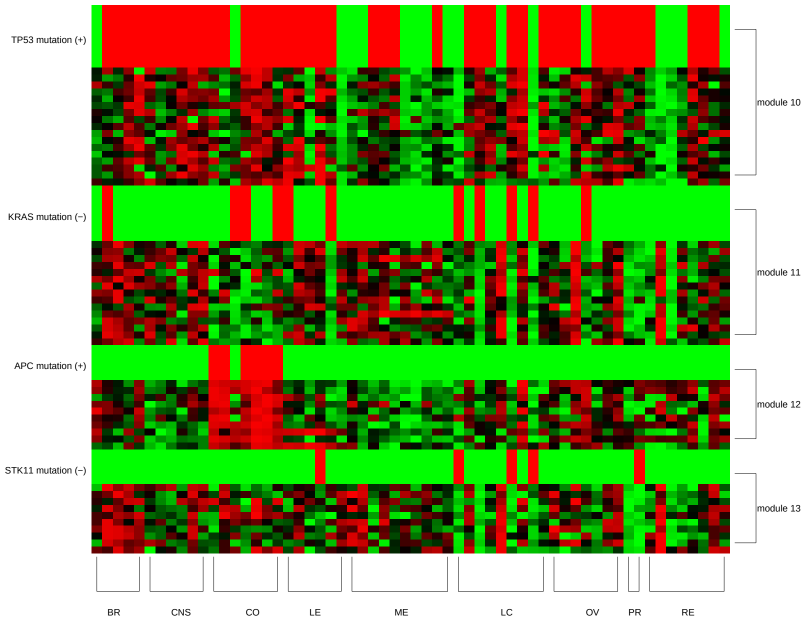

Fig. 16. Driver DNA methylation, transcription factor expressions and intra-segment CNVs and passenger microRNA expressions across NCI-60 cell lines. Modules 14-25.

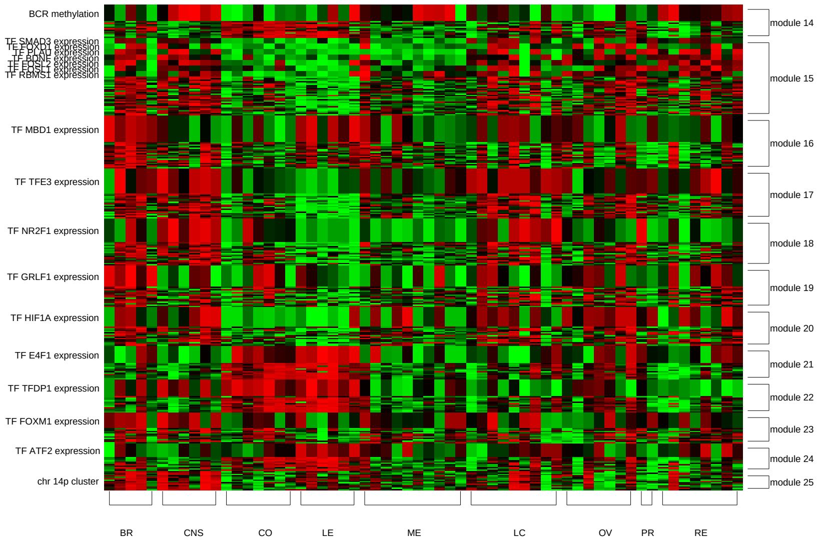

Fig. 17. Tissue-specific patterns of microRNA association modules. Each row represents the tissue-specific pattern of a module. Red: coherent up-regulation in a tissue type. Green: coherent down-regulation in a tissue type. Black: incoherent expressions in a tissue type. BRER+: ER-positive breast cancers, BRER-: ER-negative breast cancers, CNS: brain tumors, CO: colorectal cancers, LE: leukemias, ME: melanomas, LC: lung cancers, OV: ovarian cancers, PR: prostate cancers, RE: renal cancers.

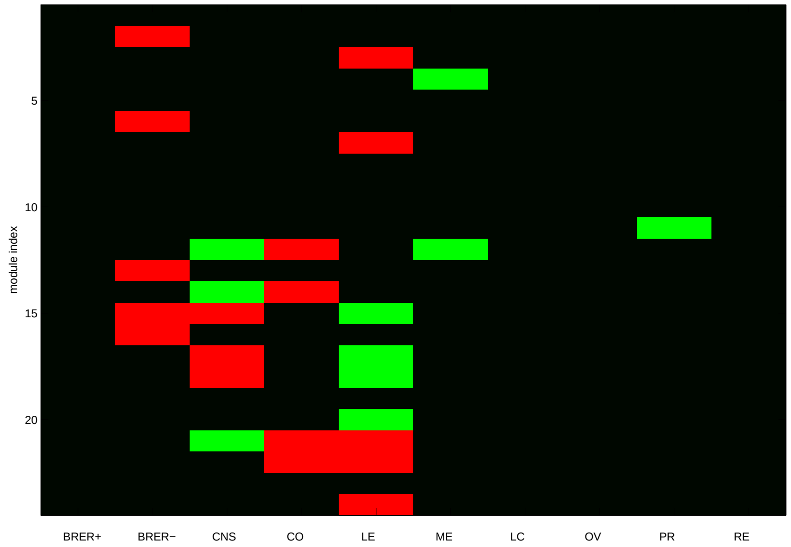

Fig. 18. Distribution of correlation coefficients between segment CNVs and their constituent genes and microRNAs. The mean of segment CNV-mRNA expressions (solid blue line) is 0.2227. The mean of segment CNV-microRNA expressions (dashed red line) is 0.0295.

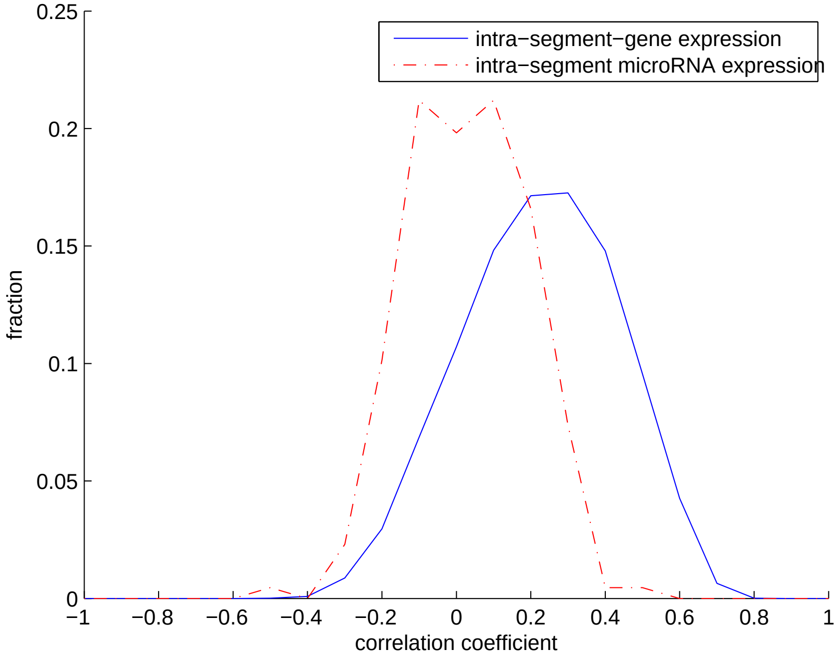

Fig. 19. NCI-60 mRNA expressions of MYB, its putative targets and control genes under normal condition and radiation treatment

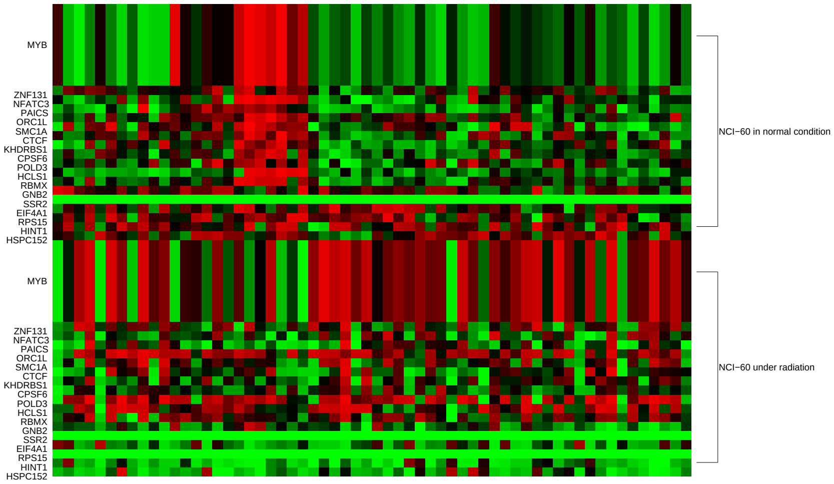

Supplement: Additional file 1 — Text S1 includes procedures of data processing, model selection, experimental and in-silico validations, heatmap visualizations of molecular aberrations and mRNA/microRNA expressions of association modules, and distributions of correlation coefficients between segment CNVs and their constituent genes and microRNAs. [file 1752-0509-5-186-S1.PDF]
